# Peripheral infrastructure vectors and an extended set of plant parts for the Modular Cloning system

**DOI:** 10.1371/journal.pone.0197185

**Published:** 2018-05-30

**Authors:** Johannes Gantner, Jana Ordon, Theresa Ilse, Carola Kretschmer, Ramona Gruetzner, Christian Löfke, Yasin Dagdas, Katharina Bürstenbinder, Sylvestre Marillonnet, Johannes Stuttmann

**Affiliations:** 1 Institute for Biology, Department of Plant Genetics, Martin Luther University Halle (Saale), Halle, Germany; 2 Department of Cell and Metabolic Biology, Leibniz Institute of Plant Biochemistry, Halle (Saale), Germany; 3 Gregor Mendel Institute (GMI), Austrian Academy of Sciences, Vienna BioCenter (VBC), Vienna, Austria; 4 Department of Molecular Signal Processing, Leibniz Institute of Plant Biochemistry, Halle (Saale), Germany; CNR, ITALY

## Abstract

Standardized DNA assembly strategies facilitate the generation of multigene constructs from collections of building blocks in plant synthetic biology. A common syntax for hierarchical DNA assembly following the Golden Gate principle employing Type IIs restriction endonucleases was recently developed, and underlies the Modular Cloning and GoldenBraid systems. In these systems, transcriptional units and/or multigene constructs are assembled from libraries of standardized building blocks, also referred to as phytobricks, in several hierarchical levels and by iterative Golden Gate reactions. Here, a toolkit containing further modules for the novel DNA assembly standards was developed. Intended for use with Modular Cloning, most modules are also compatible with GoldenBraid. Firstly, a collection of approximately 80 additional phytobricks is provided, comprising e.g. modules for inducible expression systems, promoters or epitope tags. Furthermore, DNA modules were developed for connecting Modular Cloning and Gateway cloning, either for toggling between systems or for standardized Gateway destination vector assembly. Finally, first instances of a “peripheral infrastructure” around Modular Cloning are presented: While available toolkits are designed for the assembly of plant transformation constructs, vectors were created to also use coding sequence-containing phytobricks directly in yeast two hybrid interaction or bacterial infection assays. The presented material will further enhance versatility of hierarchical DNA assembly strategies.

## Introduction

Molecular cloning belongs to the unbeloved, yet inevitable everyday tasks of many wet lab molecular biologists. In the past two decades, most labs either relied on classical ligation of restriction fragments or PCR products into a vector of interest, or used the Gateway system, which is based on the recombination reactions taking place for integration and excision of the genome of phage lambda during bacterial infection [1]. Gateway cloning proved to be extraordinarily efficient for regular cloning, and also for high-throughput applications such as library generation. One striking advantage of Gateway cloning is that only the initial creation of entry clones represents a critical step. Subsequently, inserts may be mobilized from entry clones into a wide array of destination vectors by basically failsafe, highly efficient and unified recombination reactions. This is also facilitated by the availability of destination vectors for virtually any biological system and experimental setup [e.g. 2, 3]. However, Gateway cloning is relatively costly, as it relies on use of the proprietary BP/LR enzyme blends, and also represents a rather binary approach to molecular cloning where a single insert is mobilized into a new sequence context. To some extent, this was overcome by the invention of multisite Gateway systems [4]. The current MultiSite Gateway^TM^ Pro technology allows combination of up to four DNA fragments in any (attR1/R2 site-containing) destination plasmid. Multisite Gateway was also combined with other cloning techniques in Golden GATEway cloning for further flexibility and generation of multigene constructs [5]. However, the multisite Gateway technology found only limited use in the scientific community, as novel DNA assembly strategies concomitantly emerged. Most popular strategies for combinatorial DNA assembly now rely on enzymatic reaction assembly (Gibson assembly, In-Fusion^TM^ Cloning [6–8]) or Golden Gate cloning.

In Gibson assembly [6], linear DNA fragments sharing identical sequence stretches of e.g. 20–30 base pairs at their ends are stitched together in a single tube reaction. First, 5’ ends of DNA fragments are chewed back to create single strand 3’ overhangs by an exonuclease. By the identical sequence ends, complementary fragments anneal. The annealed fragments are then covalently fused by a polymerase filling up gaps and a ligase removing nicks. Overlapping ends between fragments are also required for In-Fusion cloning. The In-Fusion enzyme generates 15-nucleotide single-stranded 5’ overhangs. Fragments anneal by complementarity, and are covalently joined after transformation in *E*. *coli*. Thus, fusion sites from enzymatic reaction assembly are scarless, no particular sequence motifs such as restriction sites are required, and large sequences up to several hundred kilobases can be assembled [6–8]. However, Gibson assembly and In-Fusion cloning rely on the engineering of identical ends on sequence fragments (by PCR), and do therefore not provide a theoretical framework for re-utilization of DNA modules in multiple and diverse DNA assemblies. This was recently achieved by the invention of hierarchical DNA assembly strategies based on Golden Gate cloning [9]. Three major standards, GreenGate [10], GoldenBraid [11] and Modular Cloning [12] were developed in parallel and are commonly used in the plant research community. All systems are based on the same principles: Standardized four base pair (bp) overhangs (generated by Type IIs restriction endonucleases) are defined as fusion sites between building blocks of transcriptional units, such as promoters, untranslated regions, signal peptides, coding sequences, or terminators. Building blocks are cloned as Level 0 modules, which are flanked by these four bp overhangs and recognition sites for a given Type IIs endonuclease. These units are also referred to as phytobricks. In a second hierarchical level (Level 1), phytobricks are assembled into transcriptional units by highly efficient Golden Gate cloning. Multigene constructs are assembled with another Golden Gate reaction and a yet further hierarchical level (Level 2 or Level M). The drawback of Golden Gate-based DNA assembly is the requirement for “sequence domestication”, the removal of internal recognition sites for respective Type IIs endonucleases from sequences of interest. Efficient strategies were previously described [9, 13], but domestication of multiple internal recognition sites may render the generation of novel phytobricks cumbersome. Also, while internal recognition sites may be eliminated through silent mutations in protein-coding sequences, consequences of domestication are hardly predictable for non-coding sequences, as e.g. promoters. Each of the Golden Gate-based assembly systems comes with its individual advantages and constraints. In GreenGate cloning, only the Type IIs enzyme *Bsa*I is used. Thus, internal recognition sites of solely this enzyme have to be removed from phytobricks during domestication. However, Level 1 vectors of the GreenGate system are not plant transformation vectors, and an additional assembly step in a Level 2 “destination” vector is thus required prior to functional verification. Furthermore, multigene construct assembly is carried out by iterative rounds where additional transcriptional units (Level 1) are added to an existing (Level 2) construct. Both GoldenBraid and Modular Cloning rely on cloning steps of different hierarchical levels being carried out by iterative use of two different Type IIs enzymes–*Bsa*I and *Bpi*I in Modular Cloning, and *Bsa*I and *BsmB*I for GoldenBraid. This obviously increases the requirement for sequence modifications during domestication, but streamlines cloning procedures and increases flexibility. The developers of GoldenBraid and Modular Cloning also agreed on a common set of fusion sites between building blocks, a common “grammar” or syntax, making these systems at least partially compatible [13, 14]. The main difference between systems consists in the approach for multigene construct assembly: Being a combinatorial process in GoldenBraid (combination of Level α and Ω), up to six Level 1 modules may be assembled in a Level 2 construct in a single step by Modular Cloning. This strategy facilitates and/or accelerates the assembly of multigene constructs, but comes at the expense of a more complex nomenclature and vector toolkit. GoldenBraid developers also provide online databases and software suites for end-users [15], and similar tools are yet unavailable for Modular Cloning. A number of research laboratories recently agreed on the use of the common molecular syntax underlying both Modular Cloning and GoldenBraid to foster re-utilization and sharing of DNA modules for bioengineering [16].

The previously released Modular Cloning Toolkit provides DNA modules facilitating domestication of novel sequences and assembly of multigene constructs following the Modular Cloning standard [13]. A simultaneously released collection of Plant Parts contains 95 modules coding for commonly used promoters, transcriptional terminators, epitope tags and reporter genes [13]. Together, these toolkits allow for a jump start into hierarchical DNA assembly for end users. In principle, the cloning of *your favorite gene* (*YFG*) in the Modular Cloning format (as a CDS1 or CDS1ns module: *YFG* flanked by *BsaI* restriction sites producing respective overhangs) will be sufficient for assembly of *YFG* together with different Plant Parts in various simple or complex plant transformation constructs. However, a peripheral infrastructure which allows re-using the Modular Cloning *YFG* modules (CDS1 or CDS1ns Level 0 modules) in other experimental setups, such as e.g. bacterial or yeast expression, and also an interface to Gateway cloning strategies, were so far missing. Here, we present molecular tools for connecting the Modular Cloning system with Gateway cloning, either for toggling between Modular Cloning and Gateway cloning, the cost-efficient generation of Gateway entry clones, or simple, hierarchical assembly of Gateway destination vectors. Furthermore, vectors were developed for re-utilization of Modular Cloning *YFG* modules for yeast two hybrid assays or bacterial translocation into plant cells. Finally, an extended collection of Plant Parts consisting of 82 Level 0 modules, or phytobricks, is provided for the sake of efficient bioengineering through shared resources.

## Material and methods

### Plant material, growth conditions, bacterial infection assays and virus induced gene silencing

*Nicotiana benthamiana* wildtype, *eds1a-1* mutant plants [17] and *pBs3*:*Bs3* transgenic plants [18] were cultivated in a greenhouse with 16 h light period, 60% relative humidity at 24/20°C (day/night). For transient *Agrobacterium*-mediated expression, plate-grown bacteria were resuspended in Agrobacterium infiltration medium (AIM; 10 mM MES pH 5.7, 10 mM MgCl_2_) to an OD_600_ = 0.4 or as indicated, and infiltrated with a needleless syringe. For imaging of IQD8 and Calmodulin2, *Agrobacterium* strains were mixed in a 1:1 ratio with a strain for expression of p19. Plasmids were mobilized into a *Pseudomonas fluorescens* strain containing a chromosomally-encoded *Pseudomonas syringae* type III secretion system ["EtHAn"; 19] by triparental mating, and plate-grown bacteria were resuspended in 10 mM MgCl_2_ prior to infiltration. For virus induced gene silencing, *Agrobacterium* solutions were infiltrated in the bottom leaves of three week-old plants. Photo-bleaching was documented 14 d later, or plants were used for challenge inoculations.

### Yeast two hybrid assays, immunoblotting and live cell imaging

Derivatives of pGAD and pGBK vectors (pJOG417-418 and pCK011-pCK012) were co-transformed into frozen competent yeast cells of strain PJ69-4a as previously described [20]. Single colonies were cultivated in liquid SD media for 48 h, and dilution series prepared. Yeast cell solutions were plated on selective media using a multichannel pipette, and plates were grown for 3–4 days prior to documentation. For immunoblot detection from yeast, proteins were extracted as previously described [21]. For extraction of plant proteins, leaf discs were ground in Laemmli buffer and boiled at 92°C for 5 minutes. Proteins were separated on SDS-PAGE gels, transferred to nitrocellulose membranes, and detected via HRP-conjugated secondary antibodies (GE Healthcare) using Supersignal West Pico and Femto substrates (Pierce; supplied by Thermo Scientific). Primary antibodies used were α-GFP (mouse monoclonal), α-HA (rat monoclonal; both from Roche and now distributed by Sigma), α-AD (GAL4 activation domain) and α-BD (GAL4 DNA-binding domain; both mouse monoclonal; Takara). Imaging was performed either on a Zeiss LSM 700 inverted microscope using a 40x water immersion objective, or a Zeiss LSM780 system. For imaging of IQD8 and Calmodulin2, mCherry was excited with a 555 nm laser, and emission was detected between 560 and 620 nm. Images are maximum intensity projections of z stacks. For simultaneous imaging of mTRQ, mEGFP and mCherry, fluorophores were excited with 458, 488 and 561 nm lasers, and emission was detected between 463–482, 499–543 and 587-630nm. For simultaneous imaging of mEGFP and chlorophyll A, 488 and 633 nm lasers were used for excitation, and emission was detected between 490–517 and 656–682 nm.

### Molecular cloning

Vectors for generation of Gateway entry vectors (pJOG130-131) were generated by ligating a PCR amplicon encoding for a *ccdB* cassette and flanked by *Bsa*I sites cutting respective 4 bp overhangs into the *Asc*I/*Not*I sites of a pENTR/D derivative. Gateway modules (pJOG267, 387, 947, 956) were generated by ligation of an attR1-ccdB/cat-attR2 PCR amplicon into pAGM1287, pICH41308 or pAGM9121 [13], respectively, or into the *EcoR*V site of a custom cloning vector (pJOG397) for generation of pJOG562. For generation of GAL4-based yeast two hybrid vectors (pJOG417-418), *Bsa*I sites in the backbones of pGAD and pGBK vectors (Clontech) were eliminated by mutagenesis, and a lacZ cassette was subsequently ligated into the *EcoR*I*/Xho*I sites. The pCK011-12 vectors were derived from these by replacing the lacZ cassette by a *ccdB* cassette with respective adaptors. The bacterial secretion vectors are based on a Golden Gate-compatible pBRM derivative [22], and secretion signals and *ccdB* cassette were ligated into the *Bsa*I*/EcoR*I sites. To generate pRNA2-GG, PCR amplicons of the 5’ and 3’ fragments of TRV2 and a *ccdB* cassette were cloned between 35S promoter and terminator sequences in pVM_BGW [23]. All Level 0 modules (S3 Table) were constructed as described [13], and internal restriction sites eliminated. For ligations or Golden Gate reactions, generally 20 fmole of all components were used, and reactions performed as previously described [12]. Primer sequences are provided in S1 Table, and additional details are available upon request.

## Results and discussion

### Golden Gate cloning vectors for Gateway entry clone generation or shuttling from Modular Cloning to Gateway cloning

**Fig 1:**
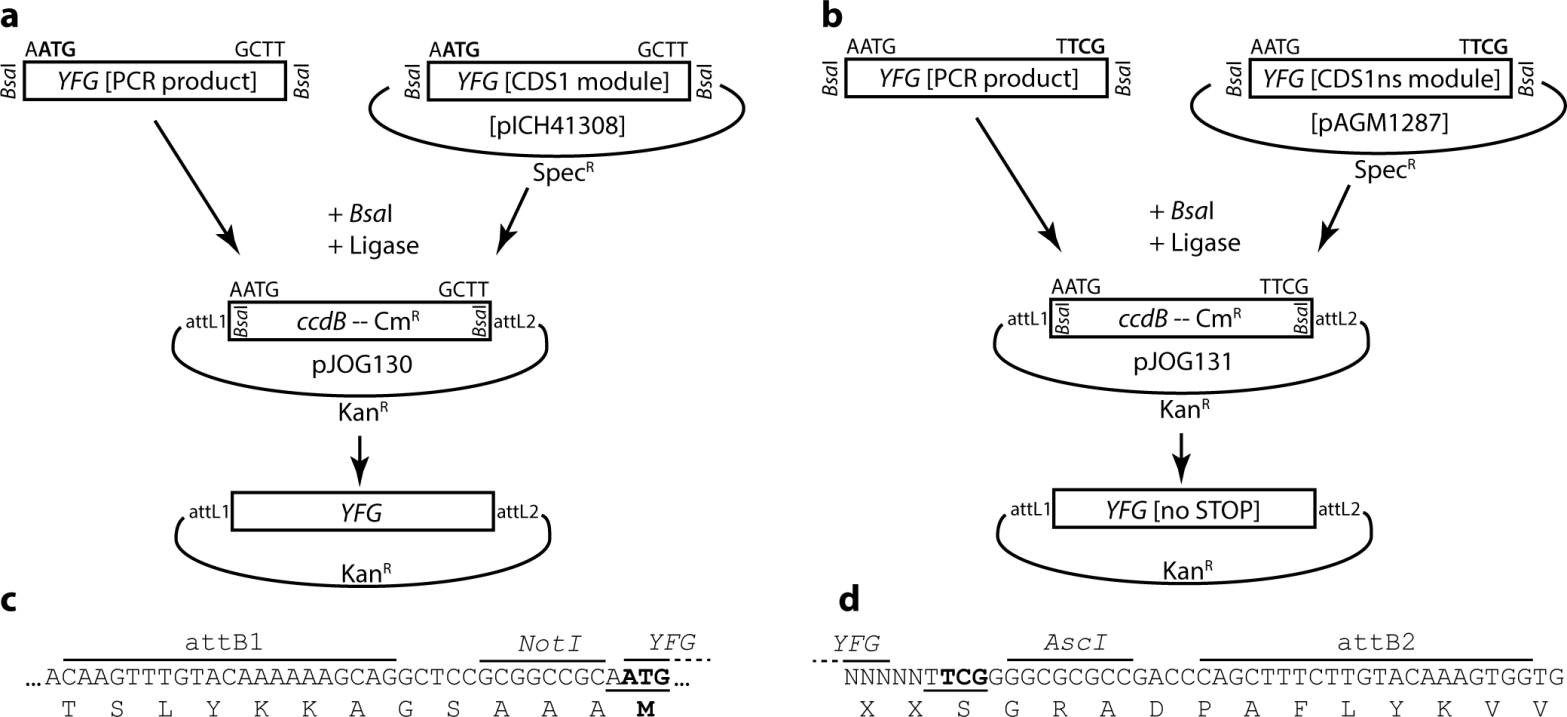
Generation of Gateway entry vectors by Golden Gate cloning. (a) Scheme of entry clone generation in pJOG130. Either PCR products flanked by *Bsa*I restriction sites and suitable 4 bp overhangs or CDS1 Level 0 modules of the Modular Cloning system may be cloned into pJOG130 by *Bsa*I cut/ligation in exchange for a *ccdB* cassette. (b) as in (a), but when using vector pJOG131 for PCR products with suitable adaptors or CDS1ns modules of the Modular Cloning system. (c) Amino acid sequence encoded by att1 sites / adaptor sequences in pJOG130. The sequence created from using a pJOG130 derivative in a LR recombination reaction is shown. Translation will either initiate at an upstream START codon of an N-terminal epitope tag, or at the ATG codon depicted in bold if no N-terminal tag is fused during LR recombination. (d) as in (c), but when using a pJOG131 derivative during LR recombination. Amino acid sequences encoded at 5’ fusion sites (attB1) are equivalent as in (c) upon fusion of an N-terminal tag. The 3’ fusion site and respective linker sequences are shown. Sequences preceding the TCG (Ser) triplet depicted in bold, which is part of the Golden Gate overhang, will depend on design of PCR product or Level 0 module.

The Gateway cloning system is widely used, and will co-exist in most labs implementing hierarchical DNA assembly strategies (in the following Modular Cloning) at least for a transitional period. For Gateway cloning, no sequence domestication is required, which might also make it the preferable system when large numbers of candidate genes are handled. Vectors were developed to ensure gene flow between cloning platforms, and also to apply the principles and nomenclature of Modular Cloning for cost-efficient Gateway entry clone generation (Fig 1). The vectors pJOG130 and pJOG131 are based on a common backbone (Kanamycin resistance, M13fwd/rev priming sites), and contain a *ccdB* negative selection cassette flanked by Golden Gate cloning sites (*Bsa*I) and attL1/2 sites. The two vectors differ in overhangs generated by *Bsa*I digestion: pJOG130 uses overhangs of CDS1 modules of Modular Cloning, while pJOG131 uses those of CDS1ns modules (Fig 1A and 1B) [12, 13]. Vectors may thus be used to convert respective modules from Modular Cloning to Gateway cloning (Level 0 -> GW entry) by simultaneous restriction and ligation using *Bsa*I (Fig 1A and 1B; in the following referred to as “*Bsa*I Golden Gate reaction”). Alternatively, PCR products carrying suitable adaptors (S1 Fig) may be cloned. While both vectors can theoretically receive PCR products containing or not a STOP codon, we intended to use pJOG130 for cloning of coding sequences with, and pJOG131 for sequences without a STOP codon to follow the Modular Cloning nomenclature. Fusion sites resulting from recombination of inserts from pJOG130 and 131 into Gateway expression vectors are depicted in Fig 1C and 1D. *Att* site-flanking *Asc*I and *Not*I sites present in most entry plasmids are maintained, and fusion sites from Golden Gate cloning translate into serine or alanine residues commonly employed as linkers.

The described vectors, pJOG130/131, were used to convert Modular Cloning Level 0 modules to Gateway entry clones, and also for cloning of ~ 60 cDNAs encoding candidate interactors obtained in a yeast three hybrid screen (to avoid sequence domestication prior to further confirmation of interactions). Toggling from Modular Cloning to the Gateways system by a *Bsa*I Golden Gate reaction was highly efficient, as previously described [9], and background free due to *ccdB* counter-selection. The efficiency of cloning PCR products depended on the quality of the PCR product and the number of internal *Bsa*I sites. Amplicons without internal *Bsa*I sites could be cloned with high efficiencies (> 80% correct clones), and also low abundance PCR products yielded reasonable efficiencies (> 20%). For cloning of amplicons with internal *Bsa*I sites, a second ligation step is required subsequent to the Golden Gate reaction [9]. Even with two internal *Bsa*I sites, cloning efficiencies from 20–80% were regularly obtained when using high-quality PCR products. It should be noted that, in rare cases, overhangs created by *Bsa*I restriction at internal sites may match vector overhangs of pJOG130/131. In these cases, and also with inserts containing >2 internal *Bsa*I sites, alternative methods for entry clone generation, such as BP reaction or TOPO cloning [1], will be preferable. Summarizing, next to shuttling inserts from Modular Cloning (or GoldenBraid) to Gateway cloning, pJOG130/131 are intended for generation of novel Gateway entry clones from PCR amplicons (containing ≤ 2 internal *Bsa*I sites) with a generalized and cost-efficient cloning strategy (< 1 € per reaction).

### Standardized assembly of simple or multipartite Gateway destination vectors by Modular Cloning

Most labs relying on the Gateway cloning strategy dispose of a rich collection of destination vectors, and many different vector series are available to the community [e.g. 2, 24, 25]. Nonetheless, e.g. the integration of improved fluorophores or specialized demands eventually necessitate the generation of novel destination vectors, which is often carried out by cumbersome and inefficient cloning strategies. However, Gateway destination vectors may also be generated by hierarchical DNA assembly from phytobricks [14].

**Fig 2:**
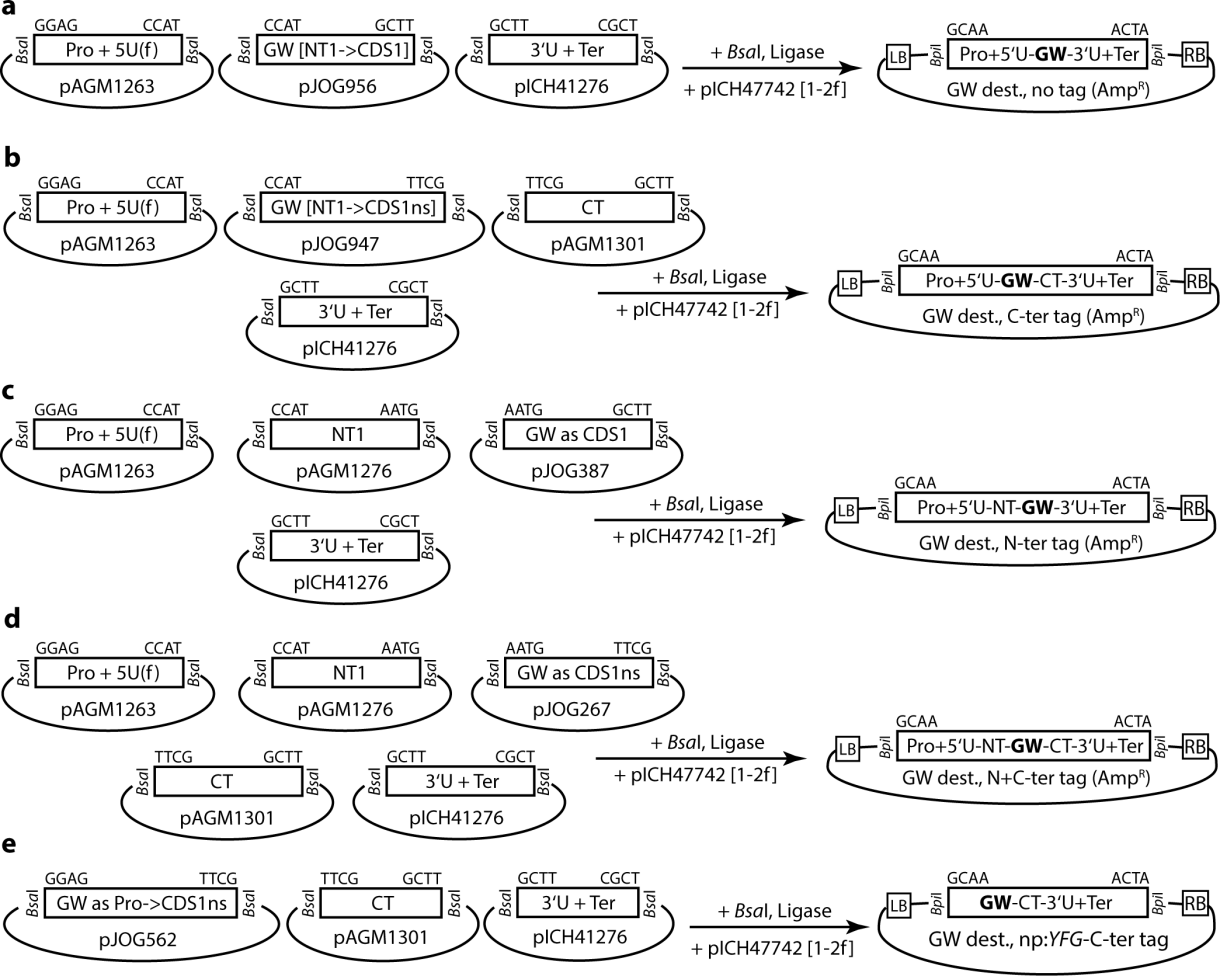
Assembly of simple Gateway (GW) destination vectors by Modular Cloning. (a) Assembly of Gateway destination vectors not encoding for epitope tags. (b) Assembly of Gateway destination vectors for C-terminal tagging of proteins of interest. (c) Assembly of Gateway destination vectors for N-terminal tagging of proteins of interest. (d) Assembly of Gateway destination vectors for N- and C-terminal tagging of proteins of interest. (e) Assembly of Gateway destination vectors for recombination of entry fragments containing both upstream regulatory sequences and a gene of interest, and for expression of C-terminally tagged proteins.

In Modular Cloning, Level 0 modules (phytobricks) are combined to a transcriptional unit in a respective Level 1 recipient [12, 13]. Five different Level 0 modules (pJOG267/387/562/947/956) containing the Gateway cassette (attR1-cat/ccdB-attR2) were constructed, and are sufficient for assembly of Gateway destination vectors for virtually any application following the standardized Modular Cloning grammar (Fig 2). It should be noted that the 5’ overhang of the Level 0 CDS1 and CDS1ns modules (A|ATG) encompasses a translation initiation codon. Thus, use of Gateway cassette-containing phytobricks of these types (pJOG267/387) in assemblies without an N-terminal tag module (NT1) will lead to a modified N-terminus in final expression products. Therefore, modules containing the NT1 5’ overhang (CCAT) and either CDS1 (pJOG956) or CDS1ns (pJOG947) 3’ overhangs were generated for assembly of Gateway destination vectors without epitope tag-encoding sequences or for C-terminal tagging, respectively (Fig 2A and 2B). pJOG387 and pJOG267 replace CDS1 and CDS1ns modules in Level 1 assemblies, and are designed for the generation of destination vectors for N-terminal or N- and C-terminal tagging of proteins (Fig 2C and 2D). Finally, pJOG562 carries overhangs to replace promoter, 5’UTR and a CDS1ns module in assembly reactions (Fig 2E). Here, assembly yields Gateway destination vectors without promoter, designed for recombination of fragments encompassing promoter and coding sequence from a respective entry clone by LR reaction.

**Fig 3:**
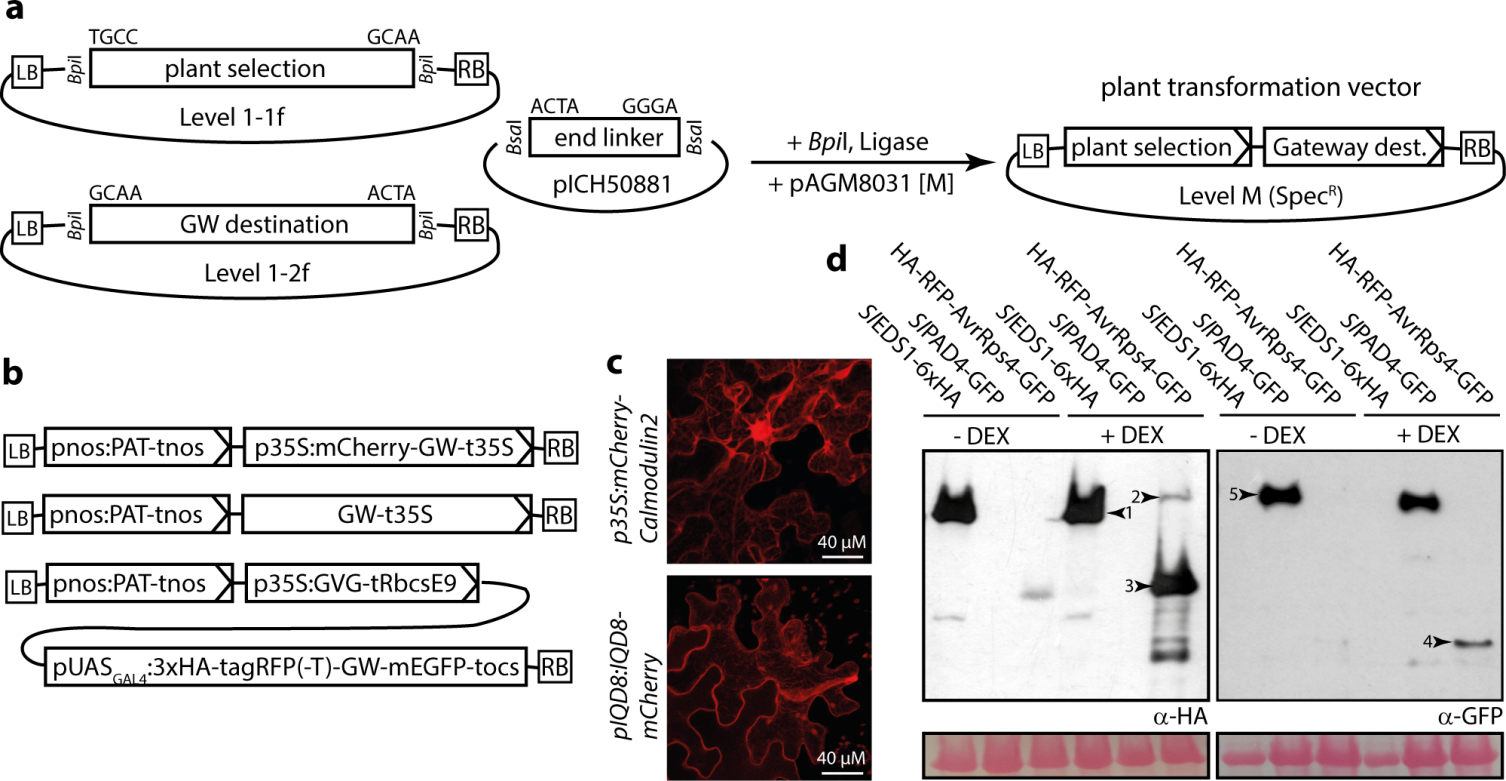
Assembly of Gateway (GW) plant transformation vectors by Modular Cloning and functional verification. (a) Exemplary scheme for assembly of a multipartite plant transformation vector containing a plant-selectable marker and a Gateway destination cassette. (b) Bi- and tri-partite plant transformation vectors assembled for functional verification. (c) Live-cell imaging of proteins transiently expressed in *N*. *benthamiana* from bi-partite transformation vectors shown in (b). Arabidopsis Calmodulin2 and IQD8 were expressed as fusions to mCherry, as indicated. Infiltrated leaf sections were analyzed 3 dpi. Maximum intensity projections are shown. (d) Immunoblot detection of proteins for functional verification of inducible expression vector shown in (b). Bands corresponding to the expected sizes of EDS1-HA (1), unprocessed HA-RFP-AvrRps4-GFP (2), processed HA-RFP-AvrRps4^N^ (3) and AvrRps4^C^-GFP (4) and PAD4-GFP (5) are marked by arrowheads.

A Level 1 assembly of the Gateway cassette-containing Level 0 modules (pJOG267/387/562/947/956) with additional phytobricks yields simple Gateway destination plasmids lacking a plant-selectable marker, which may be used e.g. for *Agrobacterium*-mediated transient expression (“Agroinfiltration”). Multipartite Gateway destination plasmids integrating a plants-selectable marker and/or additional expression cassettes are obtained by an additional assembly step (Fig 3A; Level M assembly is preferable to avoid identical resistances between entry (often Kanamycin) and destination vectors). To test efficiency and functionality of assemblies, multipartite Gateway destination vectors containing a glufosinate (BASTA) resistance cassette and for expression of mCherry fusions [26] or Dexamethasone- (DEX) inducible expression [27] were generated (Fig 3B). The DEX system relies on expression of an artificial transcription factor (GAL4-VP16-Glucocorticoid receptor, GVG), which is retained in the cytoplasm through association with Hsp90 in absence of DEX [28]. In presence of DEX, this activator binds to the synthetic promoter (pUAS_GAL4_) of a “response element” to induce expression of the gene of interest. To rebuild the DEX system [27], required components were modularized. Subsequently, the GVG expression cassette and a Gateway-compatible response element for expressions of fusions with an N-terminal 3xHA-tagRFP(-T) [29] and a C-terminal GFP tag were assembled (Fig 3B). Golden Gate reactions were highly efficient as previously described [12], and all colonies analyzed for Level 1 or Level M assemblies were correct.

**Fig 4:**
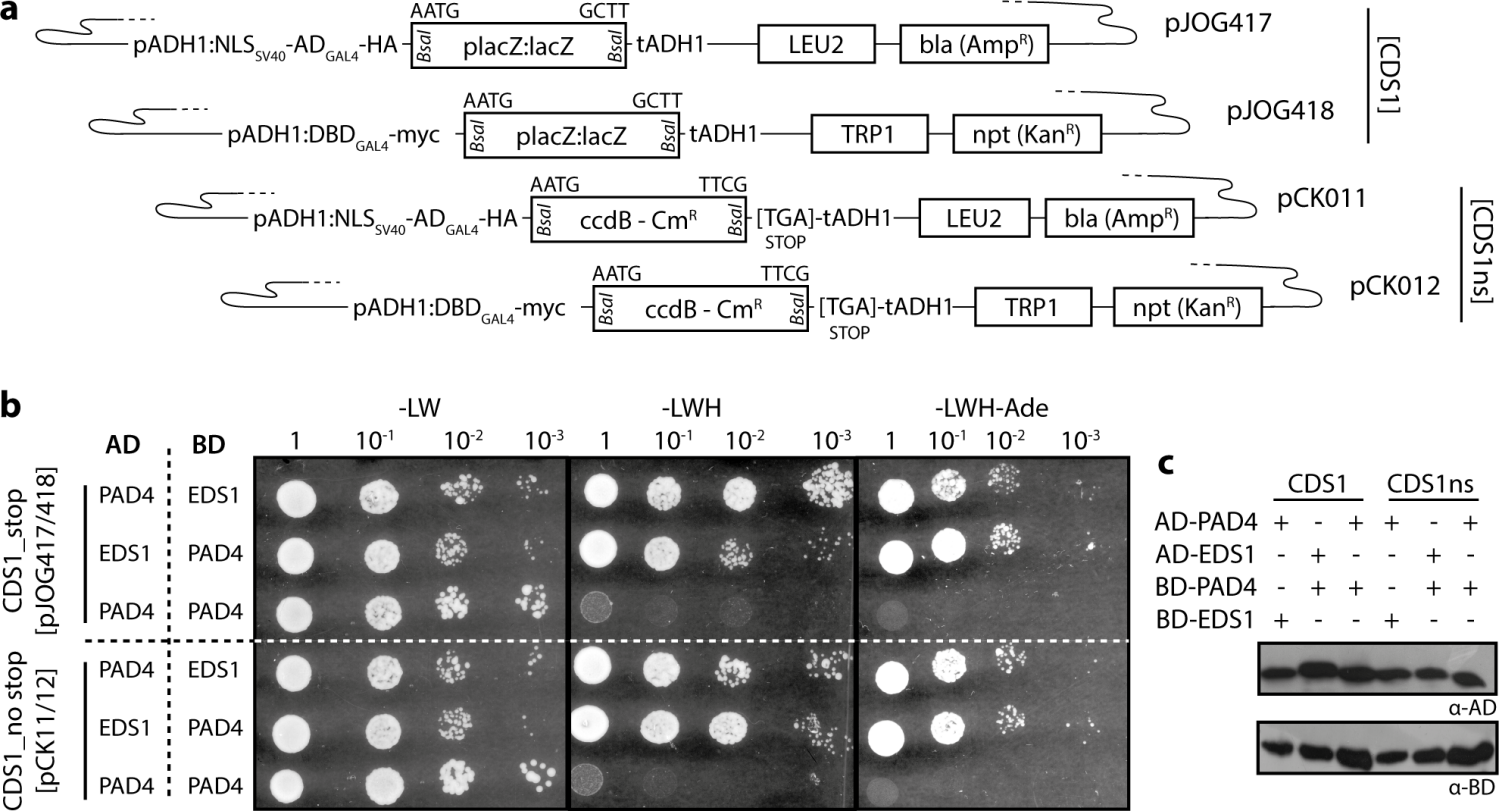
Modular Cloning-compatible vectors for a GAL4-based yeast two hybrid system. (a) Schematic depiction of yeast two hybrid vectors with most important features. (b) Functional verification of vectors shown in (a). Tomato *EDS1* and *PAD4* were mobilized into vectors shown in (a), resulting constructs co-transformed into yeast cells in the indicated combinations, and co-transformants grown in dilution series on media lacking leucine and tryptophan (-LW), or additionally lacking histidine (-LWH) and adenine (-LWH-Ade). (c) Immunoblot-detection of fusion proteins expressed in yeast cells in (b).

Calmodulin2 (under p35S control) and IQ67 domain 8 (IQD8; under native promoter control) were transiently expressed in *Nicotiana benthamiana* (*Nbenth*) leaf tissues by Agroinfiltration to test functionality of mCherry fusion vectors (Fig 3C). The newly assembled vectors, in contrast to previously tested DsRed fusion constructs, facilitated reliable live cell imaging of calmodulin2 and IQD8, in the cytosol and nucleus, and at the plasma membrane and microtubule cytoskeleton, respectively (Fig 3B and 3C)[30]. The reconstructed DEX system was tested by transiently expressing AvrRps4 from *Pseudomonas syringae* pv. *pisi* in *Nbenth* [31]. AvrRps4 is cleaved *in planta* by a yet unknown plant protease [32], which should lead to release of N- and C-terminal fragments of the 3xHA-tagRFP(-T)-AvrRps4-GFP fusion protein. Tomato EDS1 and PAD4 tagged with 6xHA and mEGFP, respectively, were constitutively expressed as control proteins, and detected both in presence and absence of DEX (Fig 4D). AvrRps4 was strongly induced in presence of DEX, and N- and C-terminal fragments were detected by respective antibodies, confirming functionality of the newly constructed inducible expression vector (Fig 4D). Thus, the presented modules and strategy allows the assembly of simple or multipartite Gateway destination vectors in a highly efficient manner and following the standardized Modular Cloning grammar.

### Yeast two hybrid vectors for use with Level 0 CDS modules of the Modular Cloning standard

The Modular Cloning system is dedicated to assembly of plant transformation constructs, and hierarchical DNA assembly resources available for e.g. yeast or bacteria do unfortunately not rely on the common plant synthetic biology syntax [16, 33, 34]. Thus, additional vector modules are required to allow seamless re-utilization of CDS1 and CDS1ns modules (encoding *your favorite gene*) in different experimental systems. As first instances of such peripheral infrastructure to the Modular Cloning system, the popular pGAD and pGBK vectors (Clontech) for GAL4-based yeast two hybrid interaction assays were converted to the Modular Cloning standard (Fig 4A). Bait and prey vectors pJOG417/418 can accommodate CDS1 modules. The analogous vectors (pCK011/012) designed to receive CDS1ns modules contain a STOP codon directly following the 3’ Golden Gate cloning overhang (T|TCG). Thus, yeast fusions proteins will contain as few as 1–2 additional amino acids (depending on the design of the respective Level 0 module), and will terminate with a serine residue encoded by the TCG within the overhang. Minimal C-terminal extensions will allow (at least in most cases) use of identical CDS1ns modules e.g. for *in planta* expression with a C-terminal epitope tag and for Y2H assays. Vectors may also be used for Golden Gate cloning of PCR products carrying *Bsa*I adapters, and are partially compatible with CDS modules of GoldenBraid (identical bacterial selection markers in pJOG417/pCK011 and pUPD).

Vectors were tested using EDS1 and PAD4 from tomato. Arabidopsis EDS1 and PAD4 strongly interact to form a heterodimeric complex [35]. We had previously confirmed that tomato EDS1 and PAD4 also interacted in Y2H using Gateway-compatible pGAD/pGBK derivatives. CDS1 and CDS1ns Level 0 modules of tomato EDS1 and PAD4 were used for *Bsa*I Golden Gate reactions with the Y2H vectors, and all tested clones were positive in restriction digests. Resulting constructs were co-transformed into yeast, and primary transformants replica-plated on reporter media in dilution series (Fig 4B). All yeast strains grew on–LW media selecting for presence of both plasmids in co-transformants. Growth on–LWH and–LWH-Ade media, indicative of interaction of bait and prey proteins, was observed upon co-expression of AD/BD fusions of EDS1 and PAD4 in either orientation, but not if PAD4 was tested for self-interaction (Fig 4B), as previously observed. All fusion proteins were detected by immunoblotting (Fig 4C), confirming full functionality of the presented Y2H vectors.

### Bacterial type III secretion vectors for the Modular Cloning standard

**Fig 5:**
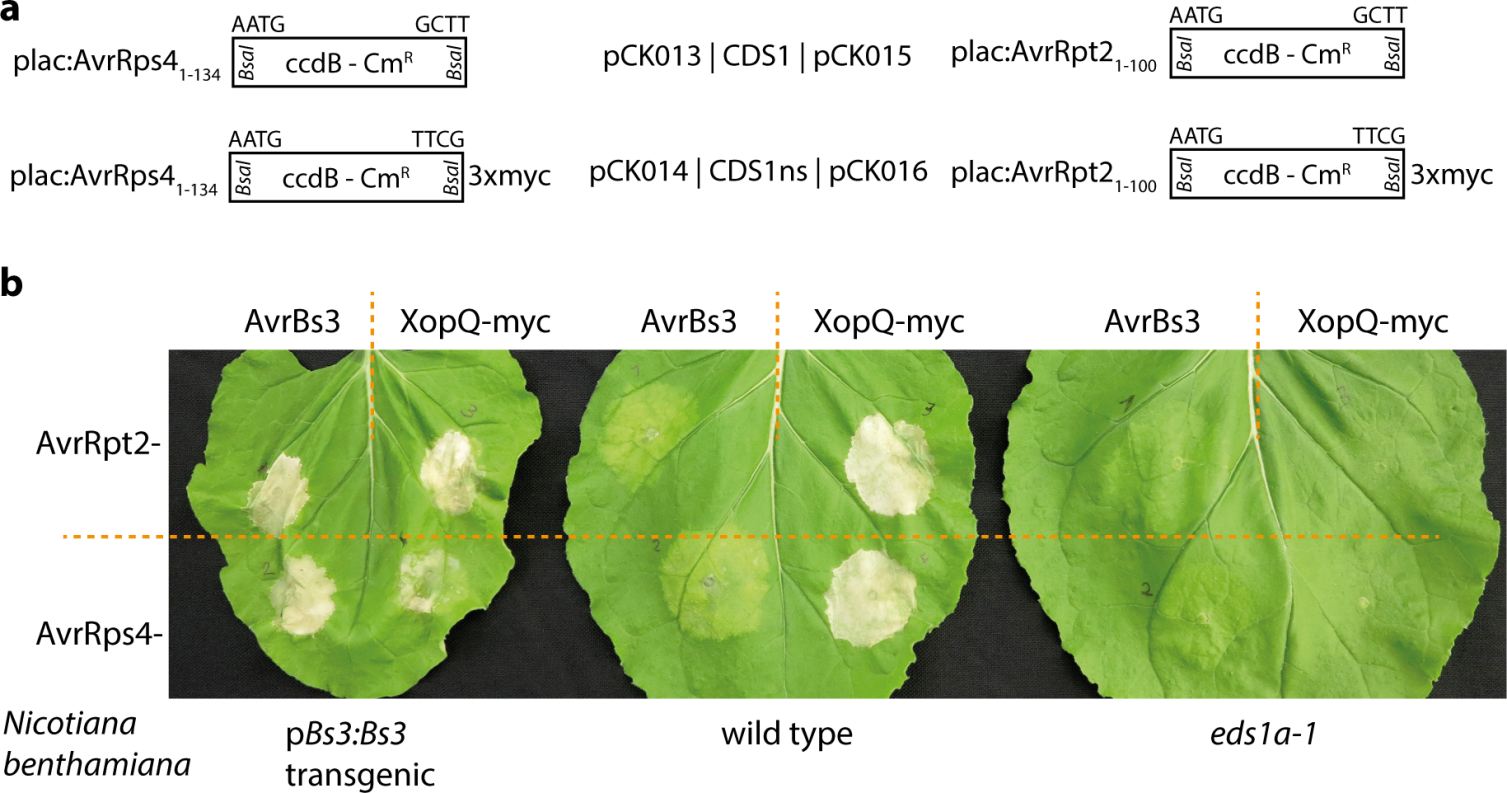
Bacterial type III-delivery of proteins into *N*. *benthamiana* cells. (a) Schematic depiction of vectors for type III-delivery of proteins. (b) Hypersensitive response induction assays for functional verification of vectors shown in (a). Either AvrBs3 or XopQ were cloned in vectors shown in (a), as indicated. Resulting constructs were mobilized into *Pseudomonas fluorescens*, strains inoculated at an OD_600_ = 0.4 on indicated *N*. *benthamiana* genotypes and symptoms documented 3 dpi.

Plant pathogenic bacteria often rely on the secretion of proteins (effectors) directly into the cytoplasm of host cells via a type III secretion system [36]. Substrates for type III secretion are recognized by a yet enigmatic N-terminal secretion signal, and proteins can be targeted for type III secretion by appending a respective signal. This has been extensively used to analyze e.g. the function of oomycete effectors in the “effector detector system” [37, 38]. Four different vectors for bacterial type III secretion and compatible with Modular Cloning (and GoldenBraid) were generated (Fig 5A). Vectors contain either amino acids 1–134 of AvrRps4 and are thus very similar to the previously described pEDV vectors [37], or amino acids 1–100 of the AvrRpt2 effector [39]. With each of these secretion signals, a vector for CDS1 modules and for CDS1ns modules was generated, and ligation of CDS1ns modules results in a C-terminal 3xmyc epitope in final fusion proteins. The *Xanthomonas euvesicatoria* genes encoding the AvrBs3 and XopQ effectors were mobilized into CDS1 and CDS1ns vectors, respectively, for functional verification. AvrBs3 is a Transcription Activator-Like Effector (TALE), and AvrBs3-mediated induction of the *Bs3* resistance gene provokes a strong and rapid cell death reaction [40, 41]. XopQ is recognized in the non-host plant *Nbenth*, and induces a mild cell death reaction [42], which is abolished on an *eds1a-1* mutant *Nbenth* line [17]. Derivatives of the bacterial secretion vectors containing AvrBs3 or XopQ were mobilized into a *Pseudomonas fluorescence* strain carrying a chromosomal integration of the type III secretion system from *Pseudomonas syringae* ["EtHAn"; 19]. Resulting strains were infiltrated into wild type, *Bs3* transgenic, and *eds1a-1* mutant *Nbenth* plants (Fig 5B). AvrBs3-expressing strains provoked strong cell death on *Bs3* transgenic plants, as expected. This confirmed that both the AvrRps4- and AvrRpt2-derived secretion signals were functional. Similarly, XopQ-expressing strains provoked cell death reactions on wild type and *Bs3* plants, but not on *eds1a-1* plants (Fig 5B). Notably, cell death reactions upon infiltration of AvrRpt2-XopQ strains were substantially and reproducibly stronger than those of AvrRps4-XopQ strains (Fig 5B and S2 Fig), suggesting that either the AvrRpt2 signal might confer higher levels of protein translocation or the respective fusion protein might be more stable or active. This demonstrates the utility of testing several different signals for bacterial translocation of a protein of interest into plant cells.

An AvrRps4_1-136_ protein fragment was previously used to mediate bacterial translocation of cargo proteins, and fusions were partially processed in plant cells due to AvrRps4 cleavage by a plant protease [37]. *In planta* processing of proteins expressed from AvrRps4_1-134_ fusion vectors presented here was not tested. Irrespective of *in planta* processing, final proteins will carry non-native N-termini and might also lack e.g. post-translational modifications, potentially impairing protein functions. Thus, we do not consider the likelihood for functionality of delivered proteins to increase through cleavage of secretion signals. Indeed, high cell death-inducing activity of AvrRpt2-XopQ, for which no cleavage is expected, demonstrates that secretion signals may have minimal and different effects on cargo functionality. To possibly avoid negative effects of the fused translocation signal on the delivered cargo moiety, the two fragments are fused by a Gly-Gly-Ser linker in pCK13-16 vectors presented here.

*Agrobacterium*-mediated expression of XopQ in wild type *Nbenth* plants induces mild chlorosis to mild necrosis [42, 43]. In contrast, bacterial translocation of XopQ here induced a strong cell death response (Fig 5B). Although XopQ recognition negatively impacts on accumulation of proteins transiently expressed by *Agrobacterium* [44], the protein accumulates to high levels in plant tissues. Also, it is generally assumed that protein levels inside the plant cell obtained by *Agrobacterium*-mediated expression largely exceed those of bacterial translocation. Increased abundance of XopQ inside plant cells upon bacterial secretion is thus not a likely explanation for the phenotypic differences. As an alternative to protein dosage, we propose that a negative effect of *Agrobacterium* strain GV3101 on HR development [45] or other constraints during transient, *Agrobacterium*-based assays [46] might be at the basis of the observed differences in XopQ-induced HR development.

### A Golden Gate-cloning vector for Tobacco Rattle Virus-induced gene silencing

**Fig 6:**
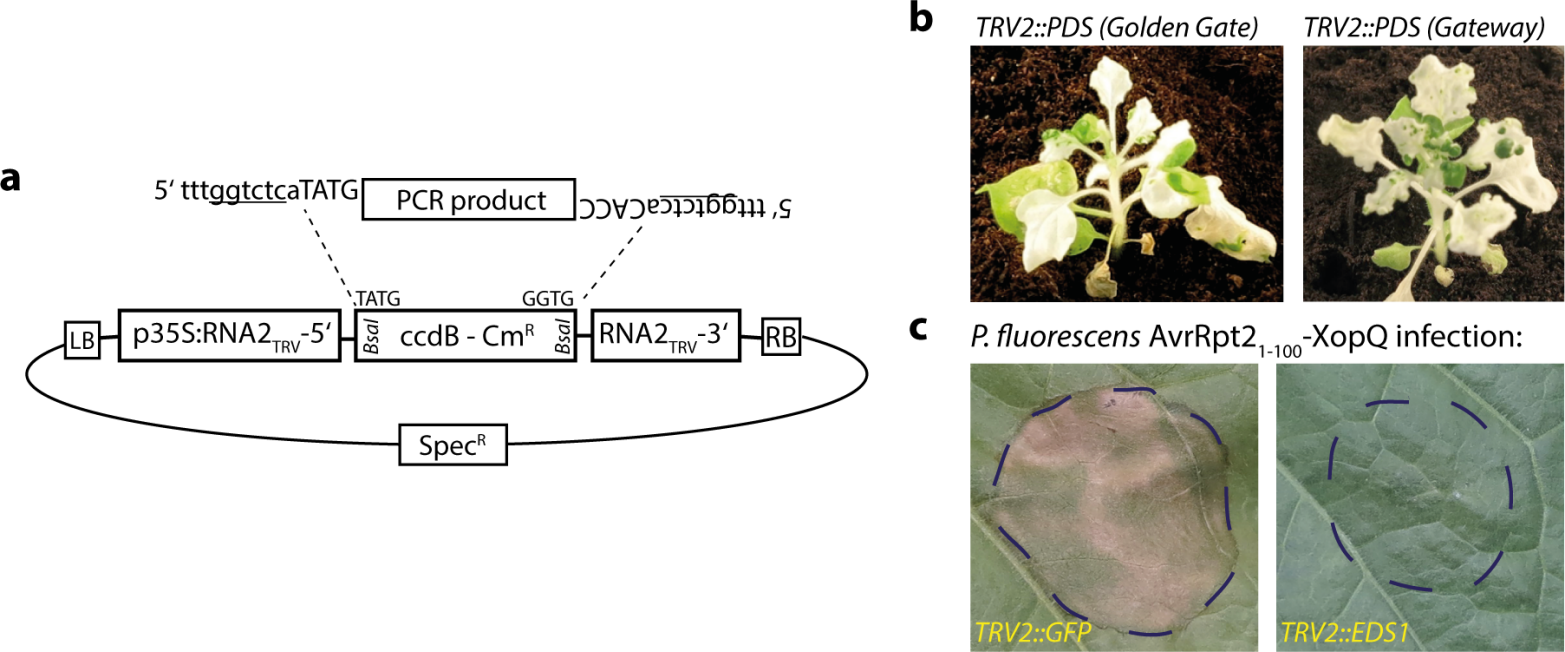
A Golden Gate cloning-compatible TRV2 vector for virus-induced gene silencing. (a) Schematic depiction of pTRV2-GG. The adaptors required for introduction of PCR products are indicated. (b) Functional verification of pTRV2-GG by silencing of *PDS*. pTRV2-GG and a commonly used Gateway-compatible TRV2 vector containing identical *PDS* fragments were compared for silencing efficiencies. (c) Silencing of *NbEDS1* using a pTRV2-GG derivative. *Pseudomonas fluorescence* bacteria expressing an AvrRpt2_1-100_-XopQ fusion protein (OD_600_ = 0.2) were inoculated 14 days after inoculation of pTRV strains, and plant reactions were documented 3 dpi.

Virus-induced gene silencing is an attractive and convenient method for the rapid knock-down of a gene of interest without the need for transformation or gene knockout. A Tobacco Rattle Virus (TRV)-based system is most commonly used, and functional in a number of different plant species including tomato and *Nbenth* [47, 48]. A fragment of the gene of interest is inserted into the RNA2 of the bipartite genome of TRV, and viral RNAs are reconstituted in the plant by expression from *Agrobacterium*-delivered T-DNAs. To facilitate rapid and cost-efficient cloning into a TRV RNA2 vector, an existing vector system [47] was adapted to Golden Gate cloning, and the cloning site was replaced by a *BsaI*-excised *ccdB* cassette as negative selection marker (Fig 6A). For functional verification, a fragment of the *Nbenth PDS* gene was inserted into a previously used, Gateway-compatible TRV2 vector and the newly generated Golden Gate-compatible vector. *PDS* encodes for Phytoene Desaturase essential for the production of carotenoids, and knock-down induces strong photo-bleaching of leaves. *Agrobacterium* strains carrying the respective TRV2 vectors were side-by-side co-inoculated with TRV1-containing strains into the lower leaves of *Nbenth* plants, and leaf bleaching documented 14 days later (Fig 6B). Both TRV vectors induced leaf bleaching to similar extents, confirming functionality of the Golden Gate-compatible derivative. The pTRV2-GG vector was also used for silencing of the *Nbenth EDS1* gene, and the XopQ-induced HR was consistently abolished on *EDS1* knock-down plants in several independent biological replicates (Fig 6C).

All vectors presented in this and previous sections are summarized in S2 Table. Annotated sequence files are provided in S1 File.

### An extended set of plant parts, or phytobricks, for the Modular Cloning system

Level 0 modules, or phyotobricks, are the building blocks and thus the limiting component for assemblies following the Modular Cloning grammar. As an extension to the previously released Plant Parts [13], we here provide ~ 80 additional Level 0 modules, summarized in S3 Table. These modules were experimentally verified as part of our ongoing projects if not indicated otherwise (S3 Table), and functional data is presented for a few selected modules. The provided new phytobricks comprise a variety of module types, e.g. modules for inducible gene expression (Fig 3), promoters for constitutive and tissue-specific gene expression in Arabidopsis (S3 Fig), transactivation (S4 Fig), additional fluorophores for (co-) localization and FRET analyses [49], signals for modifying subcellular localization, or epitope tags. In addition to the Modular Cloning and Plant Parts toolkits, these modules will further enhance the versatility of this hierarchical DNA assembly system and facilitate its implementation in the plant research community. Most provided phytobricks (~ 60) are also directly compatible with GoldenBraid (S3 Table). Described vectors (S2 and S3 Tables) will be distributed as a collection via Addgene (Kit # 1000000135), and selected vectors are also available directly through us. Annotated nucleotide sequences (GenBank format) are contained in S1 File.

## Conclusions

Novel Golden Gate-based hierarchical cloning strategies, such as Modular Cloning, allow the rapid and cost-efficient assembly of simple transcriptional units or multigene constructs from basic building blocks (phytobricks). The underlying assembly standard, or molecular grammar, ensures efficient bioengineering by re-utilization and sharing of phytobricks. Accordingly, ~ 80 novel phytobricks are provided here to foster this idea of shared resources. Furthermore, we show how the Modular Cloning assembly standard may, by integrating just a few modules, also be used for inexpensive generation of Gateway entry clones, toggling between cloning systems, or standardized assembly of Gateway destination vectors. These alternative applications of Modular Cloning may be particularly helpful to avoid the eventually laborious domestication of sequences at early stages of a project, as e.g. a first screening of candidate genes, or to connect resources available for different cloning systems.

One major advantage of Gateway cloning consists in the availability of destination vectors for virtually any biological system or experimental setup. In contrast, Modular Cloning and GoldenBraid were so far mainly designated for the generation of plant expression/transformation constructs. Similar hierarchical DNA assembly systems were developed for e.g. yeast or prokaryotes [33, 34], but only some rely on the same fusion sites between building blocks for assembly [50]. Here, we present vectors for direct use of Modular Cloning Level 0 CDS modules in yeast interaction assays or for bacterial translocation into plant cells. Similarly, new vectors need to be adapted to this cloning standard in the future, e.g. for protein production in *Escherichia coli*. This will ensure seamless and efficient integration of synthetic biology standards and novel DNA assembly strategies, and will streamline laboratory workflows by reducing molecular cloning workloads.

## Supporting information

S1 Fig Primer design for cloning into Golden Gate-compatible entry vectors pJOG130/131. (a) The *ccdB* cassette contained in pJOG130 with *Bsa*I restriction sites underlined is shown. The adaptors required for PCR amplification of suitable fragments are depicted below. Underlined sequences represent the 4 bp overhangs utilized for Golden Gate cloning, and Ns represent the gene specific portion of respective PCR primers. (b) as in (a), but for pJOG131.
(PDF)

S2 Fig Enhanced hypersensitive response induction by AvrRpt2-XopQ fusions. *Pseudomonas fluorescens* strains translocating either AvrRpt2_1-100_-XopQ or AvrRps4_1-134_-XopQ fusions were inoculated into wild type *N*. *benthamiana* plants, and symptom formation was documented 3 dpi. Four different bacterial densities, ranging from OD_600_ = 0.4–0.05 were used, and were infiltrated descendingly in the indicated leaf sections.
(PDF)

S3 Fig Promoter fragments for tissue-specific gene expression in Arabidopsis leaves. Transgenic Arabidopsis plants expressing GUS-GFP under control of the indicated promoter fragments were generated, and three-week-old T_1_ plants analyzed by confocal laser scanning microscopy. Maximum intensity projections of z-stacks are shown. Three independent T_1_ plants were analyzed for each construct with similar results.
(PDF)

S4 Fig Utilization of TALEs for tightly regulated, high-level transactivation. (a) Schematic drawing of transactivation constructs used for transient expression. (b) Strong and specific transactivation of TALE-controlled genes. *Agrobacterium* strains containing constructs depicted in (a) were infiltrated into *N*. *benthamiana*. Leaf tissues were analyzed by confocal laser-scanning microscopy 3 dpi. (c) Immunoblot analysis of protein extracts prepared from leaf tissues analyzed in (b).
(PDF)

S1 File Archive containing annotated sequence files (GenBank format) for all provided DNA modules. (ZIP)

S1 Table Oligonucleotides used in this study. (PDF)

S2 Table Modular Cloning-compatible vectors for specialized applications. (PDF)

S3 Table Modular Cloning Level 0 modules. (PDF)
