# Supplementary material for: Peripheral infrastructure vectors and an extended set of plant parts for the Modular Cloning system
Source: PLoS One. 2018 May 30;13(5):e0197185. doi: 10.1371/journal.pone.0197185 (PMC5976141; doi:10.1371/journal.pone.0197185)
Supplement: S1 Fig — (a) The ccdB cassette contained in pJOG130 with BsaI restriction sites underlined is shown. The adaptors required for PCR amplification of suitable fragments are depicted below. Underlined sequences represent the 4 bp overhangs utilized for Golden Gate cloning, and Ns represent the gene specific portion of respective PCR primers. (b) as in (a), but for pJOG131. (PDF) [file pone.0197185.s001.pdf]

Figure S1 Gantner et al.

a pJOG130

```
                                              BsaI
--GCGGCCGCAATGTGAGACC---              ---GGTCTCAGCTTGGCGCGCC--
                        cat--ccdB
--CGCCGGCGTTACACTCTGG---              ---CCAGAGTCGAACCGCGCGG--
                BsaI
```

Primer design (for cloning of CDS from ATG to STOP):

```
Forward: 5' tttggtctcaATG (N)16-20 3'

Reverse: 5' tttggtctcaaagcCTA (N)16-20 3'
                          TTA
                          TCA
```

b pJOG131

```
                                              BsaI
--GCGGCCGCAATGTGAGACC---              ---GGTCTCATTCGGGGCGCGCC--
                        cat--ccdB
--CGCCGGCGTTACACTCTGG---              ---CCAGAGTAAGCCCCGCGCGG--
                BsaI
```

Primer design (for cloning of CDS from ATG without STOP):

```
Forward: 5' tttggtctcaATG (N)16-20 3'

Reverse: 5' tttggtctcacgaagc (N)19-23 3'
                     | S | A |
```

Supplemental Figure S1: Primer design for cloning into Golden Gate-compatible entry vectors pJOG130/131.

(a) The *ccdB* cassette contained in pJOG130 with *Bsa*I restriction sites underlined is shown. The adaptors required for PCR amplification of suitable fragments are depicted below. Underlined sequences represent the 4 bp overhangs utilized for Golden Gate cloning, and Ns represent the gene specific portion of respective PCR primers.
(b) as in (a), but for pJOG131.
