# Supplementary material for: Peripheral infrastructure vectors and an extended set of plant parts for the Modular Cloning system
Source: PLoS One. 2018 May 30;13(5):e0197185. doi: 10.1371/journal.pone.0197185 (PMC5976141; doi:10.1371/journal.pone.0197185)
Supplement: S2 Fig — Pseudomonas fluorescens strains translocating either AvrRpt21-100-XopQ or AvrRps41-134-XopQ fusions were inoculated into wild type N. benthamiana plants, and symptom formation was documented 3 dpi. Four different bacterial densities, ranging from OD600 = 0.4–0.05 were used, and were infiltrated descendingly in the indicated leaf sections. (PDF) [file pone.0197185.s002.pdf]

Figure S2 Gantner et al.

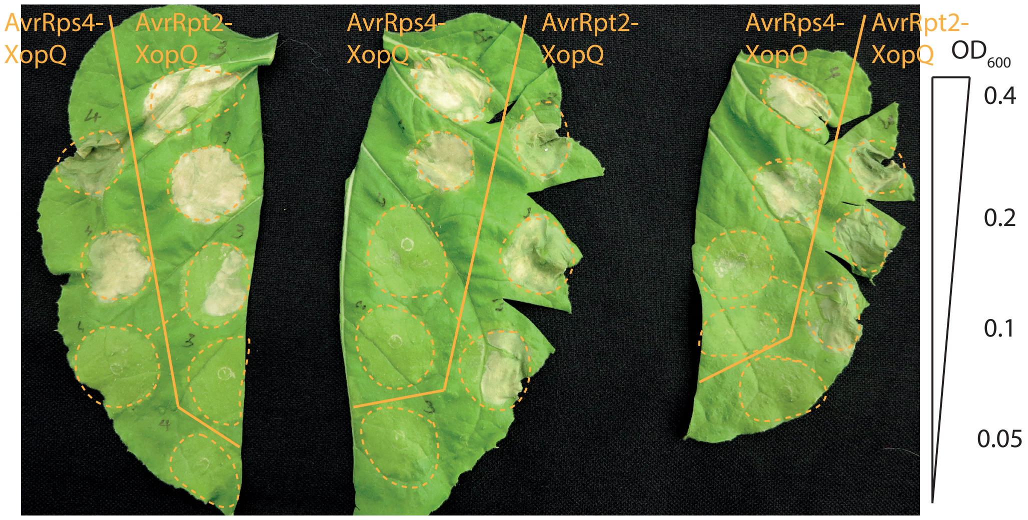

Supplemental Figure S2: Enhanced hypersensitive response induction by AvrRpt2-XopQ fusions.

Pseudomonas fluorescens strains translocating either AvrRpt2$_{1\text{-}100}$-XopQ or AvrRps4$_{1\text{-}134}$-XopQ fusions were inoculated into wild type *N. benthamiana* plants, and symptom formation was documented 3 dpi. Four different bacterial densities, ranging from OD$_{600}$=0.4-0.05 were used, and were infiltrated descendingly in the indicated leaf sections.
