# Supplementary material for: Peripheral infrastructure vectors and an extended set of plant parts for the Modular Cloning system
Source: PLoS One. 2018 May 30;13(5):e0197185. doi: 10.1371/journal.pone.0197185 (PMC5976141; doi:10.1371/journal.pone.0197185)
Supplement: S3 Fig — Transgenic Arabidopsis plants expressing GUS-GFP under control of the indicated promoter fragments were generated, and three-week-old T1 plants analyzed by confocal laser scanning microscopy. Maximum intensity projections of z-stacks are shown. Three independent T1 plants were analyzed for each construct with similar results. (PDF) [file pone.0197185.s003.pdf]

Supplemental Figure S3 Gantner et al.

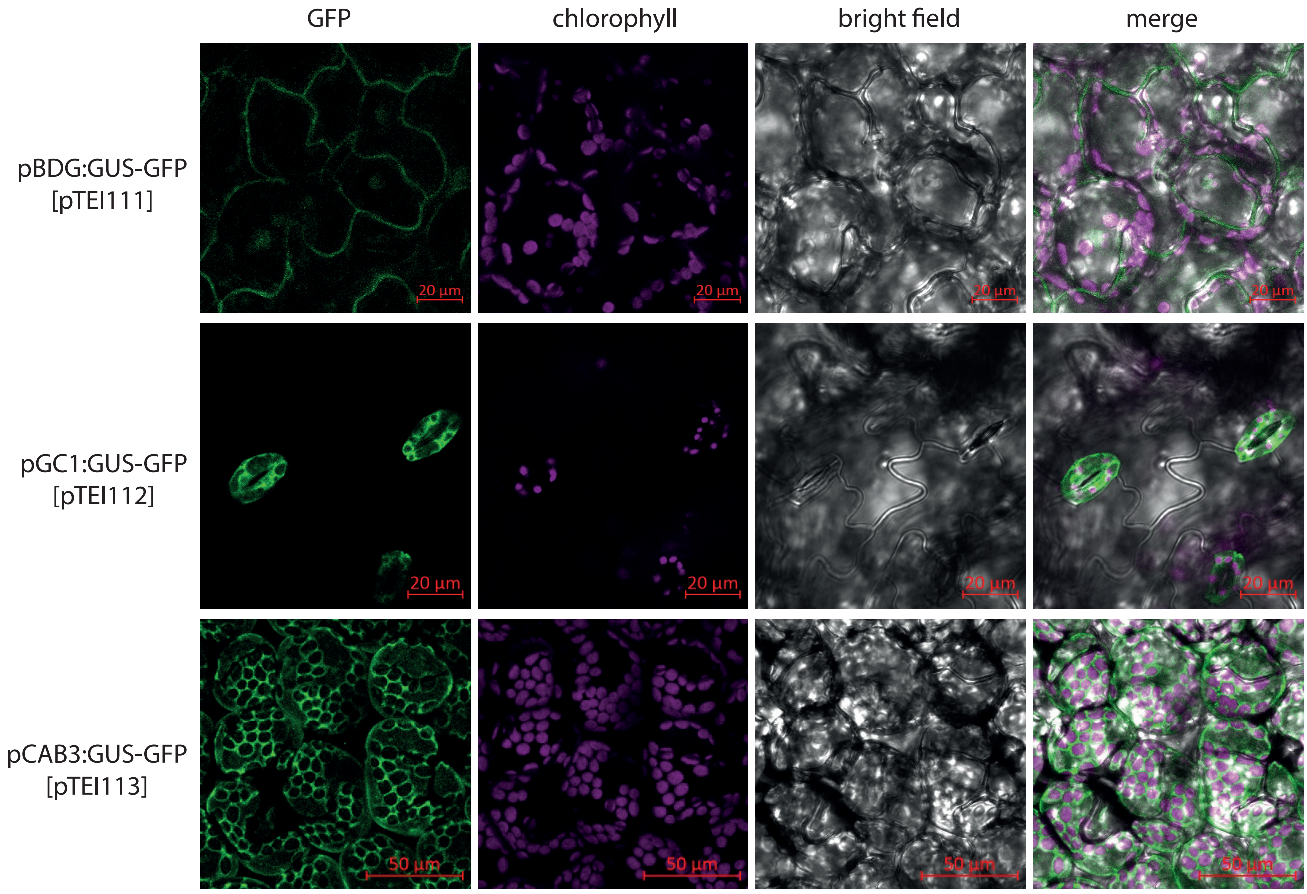

Supplemental Figure S3: Promoter fragments for tissue-specific gene expression in Arabidopsis leaves

Transgenic Arabidopsis plants expressing GUS-GFP under control of the indicated promoter fragments were generated, and three-week-old T_1 plants analyzed by confocal laser scanning microscopy. Maximum intensity projections of z-stacks are shown. Three independent T_1 plants were analyzed for each construct with similar results.
