# Supplementary material for: Peripheral infrastructure vectors and an extended set of plant parts for the Modular Cloning system
Source: PLoS One. 2018 May 30;13(5):e0197185. doi: 10.1371/journal.pone.0197185 (PMC5976141; doi:10.1371/journal.pone.0197185)
Supplement: S4 Fig — (a) Schematic drawing of transactivation constructs used for transient expression. (b) Strong and specific transactivation of TALE-controlled genes. Agrobacterium strains containing constructs depicted in (a) were infiltrated into N. benthamiana. Leaf tissues were analyzed by confocal laser-scanning microscopy 3 dpi. (c) Immunoblot analysis of protein extracts prepared from leaf tissues analyzed in (b). (PDF) [file pone.0197185.s004.pdf]

Supplemental Figure S4 Gantner et al.

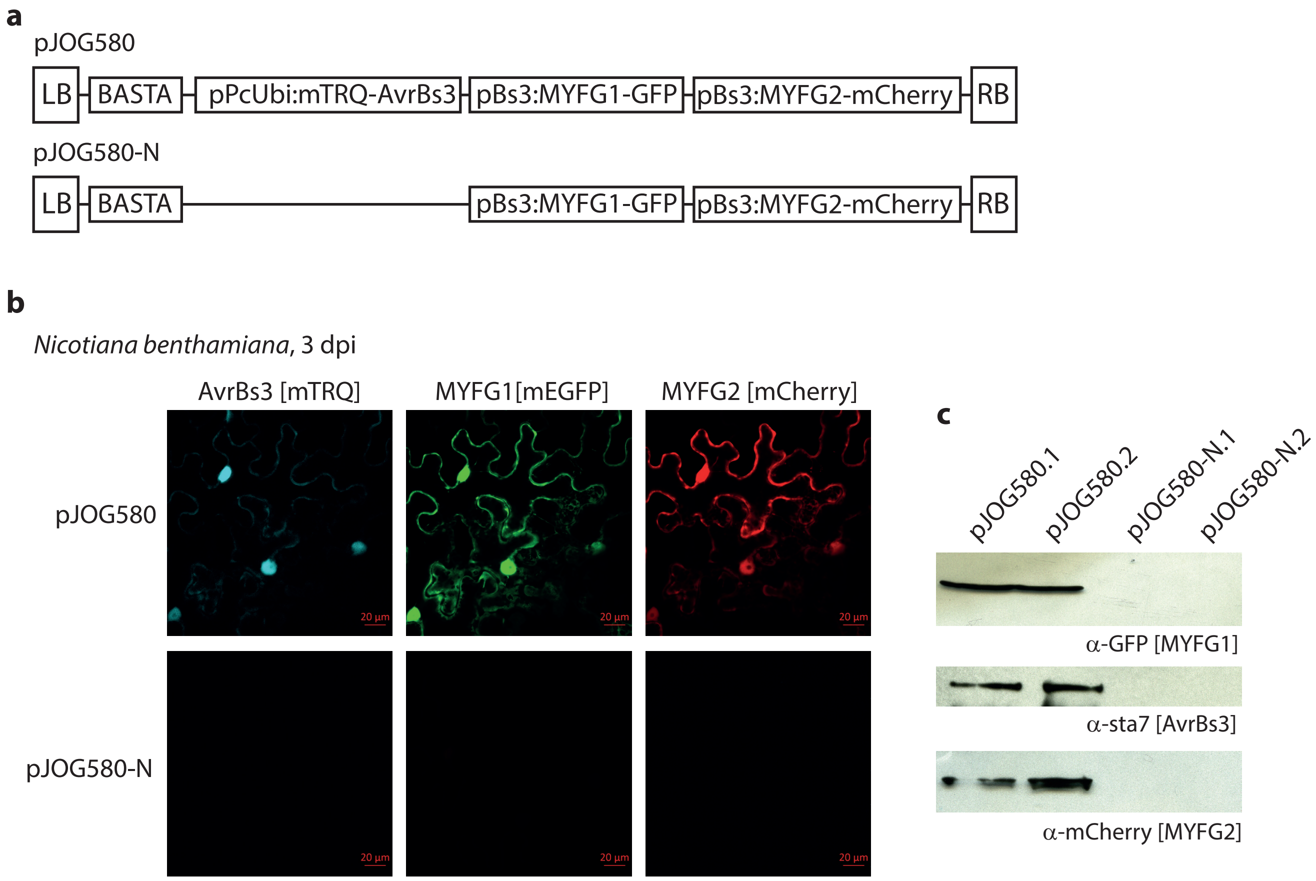

Supplemental Figure S4: Utilization of TALEs for tightly regulated, high-level transactivation

(a) Schematic drawing of transactivation constructs used for transient expression.
(b) Strong and specific transactivation of TALE-controlled genes. *Agrobacterium* strains containing constructs depicted in (a) were infiltrated into *N. benthamiana*. Leaf tissues were analyzed by confocal laser-scanning microscopy 3 dpi.
(c) Immunoblot analysis of protein extracts prepared from leaf tissues analyzed in (b).
