# Supplementary material for: Peripheral infrastructure vectors and an extended set of plant parts for the Modular Cloning system
Source: PLoS One. 2018 May 30;13(5):e0197185. doi: 10.1371/journal.pone.0197185 (PMC5976141; doi:10.1371/journal.pone.0197185)
Supplement: S1 Table — (PDF) [file pone.0197185.s006.pdf]

Table S1: Oligonucleotides used in this study

Construct	Oligo Name	Sequence
A1_pJOG130	JS946_pEGG_aatg_F	tttGCGGCCGCaatgTgagaccATTAGGCACCCCAGGCTT
	JS650-pEGG_R	tttggcgcgccaagcTGAGACCGTCGACCTGCAGACTGGC
A2_pJOG131	JS946_pEGG_aatg_F	tttGCGGCCGCaatgTgagaccATTAGGCACCCCAGGCTT
	JS963_ccdB_R	tttggcgcgccccgaaTGAGACCGTCGACCTGCAGACTGGC
A3_pJOG387	JG95_attR1_GW_F	TTTgaagacAAaatgACAAGTTTGTACAAAAAAGCTG
	JG159_GW-CDS1_R	TTTgaagacAAagcACCACTTTGTACAAGAAAGCTG
A4_pJOG267	JG95_attR1_GW_F	TTTgaagacAAaatgACAAGTTTGTACAAAAAAGCTG
	JG96_attR2_GW_R	TTTgaagacAAcgaaACCACTTTGTACAAGAAAGCTG
A5_pJOG562	JG258_GW_BsaI_F	TTTggtctcAggagACAAGTTTGTACAAAAAAGCTG
	JG259_GW_BsaI_R	TTTggtctcAcgaaACCACTTTGTACAAGAAAGCTG
A6_pJOG417	JS1143_BsaI_bla_SDM_F	GCCGGTGAGCGTGGaTCTCGCGGTATCAT
	JS1144_BsaI_bla_SDM_R	ATGATACCGCGAGAtCCACGCTCACCGGC
	JS1145_BsaI_pGAD_SDM_F	TAATACCTTCGTTGaTCTCCCTAACATGT
	JS1146_BsaI_pGAD_SDM_R	ACATGTTAGGGAGAtCAACGAAGGTATTA
	JS1170_lacZ_Y2Hvec_CDS1F	tttgaagacaaAATTaatgtgagaccAGACGGTCACAGCTTGTC
	JS1171_lacZ_pGAD_CDS1R	tttGAAGACTTtcgaAAGCtgagaccGCTGGCACGACAGGTTTC
A7_pJOG418	JS1147_BsaI_pGBK_SDM_F	AAAACCAAAAGGTCcCCGCTGACTAGGGC
	JS1148_BsaI_pGBK_SDM_R	GCCCTAGTCAGCGGgGACCTTTTGGTTTT
	JS1149_BsaI_pGBK2_SDM_F	AATACCTTCGTTGGaCTCCCTAACATGTA
	JS1150_BsaI_pGBK2_SDM_R	TACATGTTAGGGAGtCCAACGAAGGTATT
	JS1163_BsaI_pGBK3_SDM_F	ATTGGCCCCAATGGaGTCTCGGTGGGGTA
	JS1164_BsaI_pGBK3_SDM_R	TACCCCACCGAGACtCCATTGGGGCCAAT
	JS1170_lacZ_Y2Hvec_CDS1F	tttgaagacaaAATTaatgtgagaccAGACGGTCACAGCTTGTC
	JS1172_lacZ_pGBK_CDS1R	tttGAAGACTTgatcAAGCtgagaccGCTGGCACGACAGGTTTC
A8_pCK011	JS1407_ccdB for Y2HCDS1ns	tttgaagacatAATGtgagaccGACTGGCTGTGTATAAGGG
	JS1408_ccdB for Y2HCDS1ns	tttgaagacATaagcTCACGAAtgagaccTGAGACGTTGATCGGCACG
A9_pCK012	JS1407_ccdB for Y2HCDS1ns	tttgaagacatAATGtgagaccGACTGGCTGTGTATAAGGG
	JS1408_ccdB for Y2HCDS1ns	tttgaagacATaagcTCACGAAtgagaccTGAGACGTTGATCGGCACG
A10_pCK013	JS761_AvrRps4_AATT_F	tttggtctcaaattATGACTCGAATTTCAACCAG
	JS762_AvrRps4_TATG_R	tttggtctcaCATagcACCTCCACCCAATAGGGAT
	JS763_TATG_BsaI_ccdB	tttatgagagaccTGAGACGTTGATC
	JS764_ccdB-BsaI-GGTG-R	ttcacctgagaccGACTGGCTGTGTAT
	JS1180_pBRM-AvrRps4-F	TTGGGTGGAGGTgcaATGagagaccTGAG
	JS1181_pBRM-AvrRps4-R	CTCAggtctctCATtgcACCTCCACCCAA
	JS1194_pBRM-Avr-SDM	AGCCAGTCggtctcagCtTaacaaaagttgatttc
	JS1195_pBRM-Avr-SDM	gaaatcaacttttgttAaGctgagaccGACTGGCT
A11_pCK014	JS1196_pBRM-Avr-SDM	ACAGCCAGTCggtctcaTTCggaacaaaagttgatttct
	JS1197_pBRM-Avr-SDM	agaaatcaacttttgttccGAAtgagaccGACTGGCTGT
A12_pCK015	JS765_AvrRpt2_AATT_F	tttggtctcaaattATGAAAATTGCTCCAGTTGC
	JS766_AvrRpt2_TATG_R	tttggtctcaCATagagccaccGGTGGAATCGGAAGCCACG
	JS763_TATG_BsaI_ccdB	tttatgagagaccTGAGACGTTGATC
	JS764_ccdB-BsaI-GGTG-R	ttcacctgagaccGACTGGCTGTGTAT
	JS1178_pBRM-AvrRpt2-F	TCCACCggtggctcaATGagagaccTGAG
	JS1179_pBRM-AvrRpt2-R	CTCAggtctctCATtgagccaccGGTGGA
	JS1194_pBRM-Avr-SDM	AGCCAGTCggtctcagCtTaacaaaagttgatttc
	JS1195_pBRM-Avr-SDM	gaaatcaacttttgttAaGctgagaccGACTGGCT
B1_pCK016	JS1196_pBRM-Avr-SDM	ACAGCCAGTCggtctcaTTCggaacaaaagttgatttct
	JS1197_pBRM-Avr-SDM	agaaatcaacttttgttccGAAtgagaccGACTGGCTGT
B2_pTRV2-GG	JS1300_TRV5'_F	tttggtctcaattcAAGCTTGCATGCCTGCAGG
	JS1301_TRV5'_R	tttggtctcacataAGAATTCGGTAACCTTACTC
	JS1302_TRV3'_F	tttggtctcaggtgGGCCCGGGCATGTCCCGA
	JS1303_TRV3'_R	tttggtctcaaagcGTGGTGGTGGTGGTGGCT
B3_pTEI036	TI195-LexA_35Smini as Pro+5U-F	tttgaagacatggagAGCTTGGGCTGCAGGTCG
	TI196-LexA_35Smini as Pro+5U-R	tttgaagacatcatTAGAGTCGACTAGCTTCAG
B4_pTEI042	JS1339_GAl4_F	tttgaagacaaggAGCTTGCATGCCGGTCGAC
	JS1375_Gal4EBE-R	tttgaagacatCATtGGCTAGAGTCGACTAGCTTC
B5_pJOG644	JS1339_GAl4_F	tttgaagacaaggAGCTTGCATGCCGGTCGAC
	JS1340_GAL4_R	tttgaagacaaatGGCTAGAGTCGACTAGCTTC
B6_pTEI038	TI189-pG10-90 as Pro+5U-F	tttgaagacatggagATAGTTTAAACTGAAGGCGGG
	TI190-pG10-90 as Pro+5U-R	tttgaagacatcatTCCGCCCCGGAATTGGGG
B7_pJOG640	JS1352_35Sf	tttgaagacaaGGAGGAATTCCAATCCCAC
	JS1353_35Sr	tttgaagacaaATgGTATCGATAATTGTAAATG
B8_pJOG684	JS1367.2_pAtUbi-F1	tttgaagacatGGAGTGGGACCCACGGTTCA
	JS1368.2_pAtUbi-R1	tttgaagacatGACTTCGATCTAAGATTAACAG
	JS1369.2_pAtUbi-F2	tttgaagacatAGTCGATTTTCTGGGTTTGATC
	JS1370.2_pAtUbi-R2	tttgaagacatCATTTGTTAATCAGAAAAACTCAG
B9_pJOG603	JS1204_Pro5U_Bs3_F	tttgaagacaaggAGCAGCATTAAGGCACATCA
	JS1205_Pro5U_Bs3_R	tttgaagacaaCATtAAATATATGTGCAACTAGGACT
B10_pTEI044	JS1304_pRPS5a_F	tttgaagacaaggagCTCAACTTTTGATTCG

	TI203-pRPS5a as Pro+5Uf-R	
B11_pJOG301	JS1074_pDD45_F	tttGAAGACAaggagAATGGTTATAATATATAGC
	JS1075_pDD45_R	tttgaagacaaCATTATTCTTTCTTTTTGGG
B12_pAGM28521	Ec1p1	tt gaagac aa ggagcatttgcgtttggtttatcattgcg
	Ec1p2	tt gaagac aa ggacacgaaggtttaacgtcgtgttacag
	Ec1p3	tt gaagac aa gtccaataggagcgctactgattc
	Ec1p4	tt gaagac aa tagcttagtggtgatttaagagtaattaaaaag
	Ec1p5	tt gaagac aa gctagtaacgcctatcatgaattagctc
	Ec1p6	tt gaagac aa catttctcaacagattgataaggtcg
	Ec1p7	tt gaagac aa cattattctttctttttggggtttttgtttg
C1_pJOG299	JS1068_pAP1_F	tttGAAGACaaGgaGAAAACACCAAATAAGAGAAG
	JS1069-pAP1_R	tttgaagacaaCATTTTTGATCCTTTTTTAAG
C2_pJOG298	JS1055_Pro_ICU2_F	tttgaagacTTGgaGTTAATAGATGTTTAGTTTTTAT
	JS1056_Pro_ICU2_R	tttgaagacaaCATTTTTACAAATCCGGTCAA
C3_pJOG295	JS1058_GILT1_UF	tttGAAGACaaggagATGTCGCCTTAAAAAATGAATC
	JS1059_GILT1_UR	tttgaagacaaaCTCTTTTATTCGACTTTTGTC
	JS1060_GILT2_UF	tttgaagacaaGAGtCCTCACGTGAAAATGTTAC
	JS1061_GILT2_R	tttgaagacaaCATTGTTACTAAAAGAGTTTAAG
C4_pJOG300	JS1070_pAlbuminf1_F	tttGAAGACAaggaGAAACTGGTTCATCACCC
	JS1071_pAlbuminf1_R	tttgaagacaaAGtCCTCCAAAAACGAAAGAAAATTA
	JS1072_pAlbuminf2_F	tttgaagacaaGaCTCTTAGCCCTCAACTGAAAT
	JS1073_pAlbuminf2_R	tttgaagacaaCATtTCCGGAATATGGTTATA
C5_pJOG025	JG5_pAT_EDS1_1-1F	tttgaagacaaGGAGAGCTGTCTACATGAATCGAATC
	JG6_pAT_EDS1_1-1R	tttgaagacaaCgTCGTCTCTTTAATATAGACTCCA
	JG7_pAT_EDS1_1-2F	tttgaagacaaGAcGACTCCAAAGTCAAGCTACAA
	JG8_pAT_EDS1_1-2R	aaagaagacaacattGATCTATATCTATTCTCTTTTCTT
	JG23_AtEDS1prom_sdm_1f	TTCTTCTATACGAGtCCAAATCTGAAAAC
	JG24_AtEDS1prom_sdm_1r	GTTTTCAGATTTGGaCTCGTATAGAAGAA
	JG25_AtEDS1prom_sdm_2f	TTGCATTGAAATGGaCTCATGATGGGGTAT
	JG26_AtEDS1prom_sdm_2r	ATACCCCATCATGAGtCCATTTCAATGCAA
C6_pJOG123	JG5_pAT_EDS1_1-1F	tttgaagacaaGGAGAGCTGTCTACATGAATCGAATC
	JG71_pAT_EDS1_1-2R	aaagaagacaaatggGATCTATATCTATTCTCTTTC
C7_pJOG026	JG09_pAT_PAD4_1-1F	tttgaagacaaGGAGAGCAATTACAAGATTGCTTTA
	JG10_pAT_PAD4_1-1R	tttgaagacaaCcTCTTCAAAGTCTCTTACTTTAACATC
	JG11_pAT_PAD4_1-2F	tttgaagacaaGAgGACGACTTAGCAAAGACCAAAACC
	JG12_pAT_PAD4_1-2R	aaagaagacaacattATTGGATATCGAGTAGAGAGTTGCAGAAC
C8_pJOG124	JG09_pAT_PAD4_1-1F	tttgaagacaaGGAGAGCAATTACAAGATTGCTTTA
	JG72_pAT_PAD4_1-2R	aaagaagacaaatggATTGGATATCGAGTAGAGAG
C9_pJOG466	JS1185_pAtSAG101-F	tttgaagacaaggagTGACGATGGTTGTTAAACGA
	JS1186_pAtSAG101-P5U-R	tttgaagacaaCATtGCGTGAGCTGGAATAGATCTGTGGAcAAGACAATAC
C10_pJOG467	JS1185_pAtSAG101-F	tttgaagacaaggagTGACGATGGTTGTTAAACGA
	JS1187_pAtSAG101-P5Uf-R	tttgaagacaaATgGCGTGAGCTGGAATAGATCTGTGGAcAAGACAATAC
C11_pJOG010	JG1_pPR1-1_F	tttgaagacaaggagTTCATGCTAAACTATTTCTCG
	JG2_pPR1-1_R	tttgaagacaaGTCaTCTCTATTTCAAATTTGAAT
	JG3_pPR1-2_F	tttgaagacaatGACTTAAATTAGAATCATGAAG
	JG4_pPR1-2_R	tttgaagacaacattTTTCTAAGTTGATAATGGTTAT
C12_pJOG648	JS1333_At2S3Pro-F	tttGAAGACATGGaGAAGATAAATTTGCGAGTC
	JS1334_At2S3Pro-R	tttGAAGACAACATTTTTGCTATTTGTGTATGTTTTCTTG
D1_pTEI067	TI218_CER6_b_F	tttgaagacatGaCTCTTCATTAACTCCTCTC
	TI219_CER6_b_R	tttgaagacatCATtGTCGGAGAGTTTTAATGTATA
D2_pTEI068	TI210_CUE1_F	tttgaagacatGGAGTACGTAAACTCTTGT
	TI211_CUE1_R	tttgaagacatCATtGTTGAGATCTGGAATCAGAAG
D3_pTEI069	TI212_BDG_F	tttgaagacatgGAGAGAATGGTATGTTGAGTAAAG
	TI213_BDG_R	tttgaagacatCATtCTGTGGAGTTTGAGTCTTGTGcAAG
D4_pTEI070	TI214_GC1_F	tttgaagacatggAGGAATTGACGATGTAGAATTGTAG
	TI215_GC1_R	tttgaagacatCATtTTTCTTGAGTAGTGATTTTGAAG
D5_pTEI071	TI222_CAB3_Fa	tttgaagacatgGAgTCTGAAGCTCGTAACATTGGC
	TI223_CAB3_Ra	tttgaagacatAGtCCACATGTTGCAAGTCTTTTGG
	TI224_CAB3_F_b	tttgaagacatGaCTCGAAATGCTTTGGCTGC
	TI225_CAB3_R_b	tttgaagacatCATTGAAACTTTTTGTGTTTTTTTTTTTTTTTGGTG
D6_pTEI073	TI220_ATML12_F	tttgaagacatGGagCACGTTGTATCCATGAAGCT
	TI221_ATML12_R	tttgaagacatCATTTTCAGGGAGAGAAATTGG
D7_pCK019	JS1438_pEXP7_F	tttgaagacatggagCTTTGCTTTCTCCGGTTC
	JS1439_pEXP7_R	tttgaagacatCATtctagcctctttttctttattc
D8_pCK020	JS1440_Wox5-F	tttgaagacatGgaGtaaggctagacaacgtcc
	JS1441_Wox5-R	tttgaagacatCATTgttcagatgtaaagtcctc
D9_pCK021	JS1448_pSOMBR-F1	tttgaagacatggagTCGTTGAAGATGCCTGGA
	JS1449_pSOMBR-R1	tttgaagacatAcACGTCATGCATTAAACCC
	JS1450_pSOMBR-F2	tttgaagacatGTgTTCGTGAAGGCAACGGCA
	JS1451_pSOMBR-R2	tttgaagacatGtAGACAATGAAAGAGAGAGA
	JS1452_pSOMBR-F3	tttgaagacatCTaCTACCTCTTGCCTCTAACAG
	JS1453_pSOMBR-R3	tttgaagacatCATTATCCTTACTCTTCTTTAAGC
D10_pAGM1761	Dmc1	ttt gaagac aa ggagctatgagattactcgtgtatc
	Dmc2	ttt gaagac aa gaccaacaaatggctaaacaaatggc
	Dmc3	ttt gaagac aa ggtcatcatttctcatgctttgattacatg

	Dmc4	ttt gaagac aa gacacctgatcaagttcaagtttaatttg
	Dmc5	ttt gaagac aa tgtcacaatatatacaatttcgaggg
	Dmc6	ttt gaagac aa agtagctcgatttgcttcgaggg
D11_pJOG020	JS834_Parsleyubq4-2_F	tttGAAGACTTggagATTACGGATATGAATATAGGC
	JS835_Parsleyubq4-2_R	tttGAAGACTTcattGCTGCACATACATAACATATC
D12_pJOG575	JS834_Parsleyubq4-2_F	tttGAAGACTTggagATTACGGATATGAATATAGGC
	JS1252_PcUbi_R	tttgaagacaaATGGCTGCACATACATAACATATC
E1_pJOG022	JS856_UBI_F	tttGAAGACttGGAGggtcgtgcccctctctag
	JS857_UBI_R	AAAGAAGACaaCATTctgcagaagtaacaccaaac
E2_pJOG213	JS826_promSlEDS1-1_F	TTTgaagacAAggagTCTGATATGATCGGAATGATAAG
	JG87_promSlEDS1_R	TTTgaagacAAcattTTTTGCTTCTAGCTGCTTA
E3_pJOG004	JS826_promSlEDS1-1_F	TTTgaagacAAggagTCTGATATGATCGGAATGATAAG
	JS827_promSlEDS1-1_R	tttGAAGACaaCgTCCATAAGAACAAGAACAAGAG
	JS828_promSlEDS2-1_F	tttGAAGACaaGAcGACATAAAGATGCAAGTG
	JS829_promSlEDS2-1_R	TTTgaagacAAatggTTTTGCTTCTTAGCTGCTTA
E4_pJOG535	JS1204_Pro5U_Bs3_F	tttgaagacaaggAGCAGCATTAAGGCACATCA
	JS1205_Pro5U_Bs3_R	tttgaagacaaCATtAAATATATGTGCAACTAGGACT
E5_pJOG577	JS1204_Pro5U_Bs3_F	tttgaagacaaggAGCAGCATTAAGGCACATCA
	JS1253_pBs3_R	ttgaagacaaATggTAAATATATGTGCAACTAGGAC
E6_pJOG638	JS1323_SDMpBs3_F	TTATTATATAAACCgAACCATCCTCACAA
	JS1324_SDMpBs3_R	TTGTGAGGATGGTTcGGTTTATATAATAA
E7_pJOG639	JS1323_SDMpBs3_F	TTATTATATAAACCgAACCATCCTCACAA
	JS1324_SDMpBs3_R	TTGTGAGGATGGTTcGGTTTATATAATAA
E8_pJOG170	JS965_FP_BpiI_NT1_F	tttgaagacaaccATGGTGAGCAAGGGCGAGG
	JS966_FP_BpiI_NT1_R	tttgaagacAAcattgcagatTTGTACAGCTCGTCCATGC
E9_pJOG176	JS969_FP_BpiI_CT_F	tttgaagacaattcggctGTGAGCAAGGGCGAGGAGC
	JS968_FP_BpiI_CDS1_R	tttgaagacAAagcTCATTTGTACAGCTCGTCCATGC
E10_pJOG169	JS965_FP_BpiI_NT1_F	tttgaagacaaccATGGTGAGCAAGGGCGAGG
	JS966_FP_BpiI_NT1_R	tttgaagacAAcattgcagatTTGTACAGCTCGTCCATGC
E11_pJOG175	JS969_FP_BpiI_CT_F	tttgaagacaattcggctGTGAGCAAGGGCGAGGAGC
	JS968_FP_BpiI_CDS1_R	tttgaagacAAagcTCATTTGTACAGCTCGTCCATGC
E12_pJOG168	JS965_FP_BpiI_NT1_F	tttgaagacaaccATGGTGAGCAAGGGCGAGG
	JS966_FP_BpiI_NT1_R	tttgaagacAAcattgcagatTTGTACAGCTCGTCCATGC
F1_pJOG174	JS969_FP_BpiI_CT_F	tttgaagacaattcggctGTGAGCAAGGGCGAGGAGC
	JS968_FP_BpiI_CDS1_R	tttgaagacAAagcTCATTTGTACAGCTCGTCCATGC
F2_pJOG142	JG79_YFP-NES/NLS_CT_F	tttgaagacaatTCGgctATGGTGAGCAAGGGCGAG
	JG80_YFP-NES_CT_R	tttgaagacaaaagcttaaatatcaagacctgcaagtttgagagcaagCTTGTACAGCTCGTCCATGCCG
F3_pJOG143	JG79_YFP-NES/NLS_CT_F	tttgaagacaatTCGgctATGGTGAGCAAGGGCGAG
	JG81_YFP-NLS_CT_R	tttgaagacaaaagcttaAACCTTTCTCTTCTTCTTAGG
F4_pJOG140	JG77_NES_NT1_F	ccATGGCTTCTcttgctctcaaacttgcaggtcttgatattAGCgc
	JG78_NES_NT1_R	CATTgcGCTaatatcaagacctgcaagtttgagagcaagAGAAGCC
F5_pJOG141	JG75_NLS_NT1_F	ccATGGCTAGCCCACCGAAGAAGAAGCGGAAGGTCAGCgc
	JG76_NLS_NT1_R	CATTgcGCTGACCTTCCGCTTCTTCTTCGGTGGGCTAGCC
F6_pJOG824	JS1513_NLS-F	tTCGgctgcagccCCTAAGAAGAAGAGAAAGGTTTGA
	JS1514_NLS-R	aagcTCAAACCTTTCTCTTCTTCTTAGGggctgcagc
F7_pAGM16456	Primu1	tt ggtctc a ACAT ccatgggcagccatcaccaccatcatc
	Primu11	tt ggtctc a ACAA catcccgctgtgatgatggtggtgatggctgc
	Primu20	tt ggtctc a ACAT gatgctacaactcccacctctagagcgcttgac
	Primu22	tt ggtctc a ACAA catt ccgtccagtgtcaagcgctctagaggtgg
F8_pAGM16467	Primu1	tt ggtctc a ACAT ccatgggcagccatcaccaccatcatc
	Primu11	tt ggtctc a ACAA catcccgctgtgatgatggtggtgatggctgc
	Primu21	tt ggtctc a ACAT gatgctagcactgaagcttgctggcctcgac
	Primu23	tt ggtctc a ACAA catt ccgttgatgtcgaggccagcaagcttc
F9_pAGM16418	Primu1	tt ggtctc a ACAT ccatgggcagccatcaccaccatcatc
	Primu2	tt ggtctc a ACAA tccgctgtgatgatggtggtgatggctgc
	Primu16	tt ggtctc a ACAT cggactacaactcccacctctagagcgcttgac
	Primu17	tt ggtctc a ACAA catcccgtccagtgtcaagcgctctagaggtgg
	Primu5	tt ggtctc a ACAT gatggtgagcaagggcgagga
	Primu6	tt ggtctc a ACAA cattgaacccttgtacagctcgtccatg
F10_pAGM16429	Primu1	tt ggtctc a ACAT ccatgggcagccatcaccaccatcatc
	Primu2	tt ggtctc a ACAA tccgctgtgatgatggtggtgatggctgc
	Primu18	tt ggtctc a ACAT cggactagcactgaagcttgctggcctcgac
	Primu19	tt ggtctc a ACAA catcccgttgatgtcgaggccagcaagcttc
	Primu5	tt ggtctc a ACAT gatggtgagcaagggcgagga
	Primu6	tt ggtctc a ACAA cattgaacccttgtacagctcgtccatg
F11_pJOG642	JS1341_HAtag_F	tttgaagacaaCCATGTACCCATACGATGT
	JS1342_HAtag_R	tttgaagacaaACAGCAGCTCTAGTGGCGT
	JS1343_tagRFP-F	tttgaagacaactGTGTCTAAGGGCGAAGAGC
	JS1344_tagRFP-R	tttgaagacaaCcTCGTATGTGGTGACTCTC
	JS1345_tagRFP2-F	tttgaagacaaGAgGACGGGGGCGTGCTGAC
	JS1346_tagRFP2-R	tttgaagacaaCATtgcagcagaCTTGTACAGCTCGTCCATG
F12_pJOG317	JS1080_1Strep_F	tttgaagacaacgctTCTGCTTGGTCACACCCAC
	JS1081_1Strep_CT_R	tttgaagacaaaagcTATTTTTCAAATTGAGGATG

	JS1082_4xmyc_CT1_F	tttgaagacaaTTCGGAACAAAAGTTGATC
	JS1083_4xmyc_R	tttgaagacaaagcggcAAGGTCCTCTTCAGAAATAAG
G1_pJOG331	JS1078_6xHA_CT1_F	tttgaagacaaTTCGTACCCATACGATGTTC
	JS1079_6xHA_R	tttgaagacaaagcGGCGTAATCTGGAACGTC
	JS1080_1Strep_F	tttgaagacaacgctTCTGCTTGGTCACACCCAC
	JS1081_1Strep_CT_R	tttgaagacaaaagcTATTTTTCAAATTGAGGATG
G2_pJOG658	JS1341_HAtag_F	tttgaagacaaCCATGTACCCATACGATGT
	JS1342_HAtag_R	tttgaagacaaACAGCAGCTCTAGTGGCGT
	JS1362_LUC-F	tttgaagacaactgtgtctgctGAGGACGCCAAAAACATAAAG
	JS1363_LUC-R	tttgaagacaaCATtgcggcactagcCAATTTGGACTTTCCGCC
G3_pJOG659	JS1356_REN-F	tttgaagacaatTCGgctACTTCGAAAGTTTATGATC
	JS1357_REN5pr-R	tttgaagacaaAGgCCTTTTACTTTGACAAATTC
	JS1358_REN3pr-F	tttgaagacaaGcCTTCATTTTTCGCAAGAAG
	JS1359_REN3pr-R	tttgaagacaagcagaTTGTTCATTTTTGAGAACTC
	JS1360_3xHA-F	tttgaagacaactgctgccTACCCATACGATGTTCCTG
	JS1361_3xHA-R	tttgaagacaaaagctaggcAGCGTAATCTGGAACGTC
G4_pJOG643	JS1349_GR-F	tttgaagacaaccATGATTCAGCAAGCCACTGC
	JS1350_GR-R	tttgaagacaaCATtgcggcagaagcTTTTTGATGAAACAGAAGCTT
G5_pCK032	JS1454_GRasCT-F	tttgaagacatTTCGgctgcaTCTGCTATTCAGCAAGCCACTGC
	JS1455_GRasCT-R	tttgaagacTAaagcTCATTTTTGATGAAACAGAAGCTT
G6_pTEI037	JS1349_GR-F	tttgaagacaaccATGATTCAGCAAGCCACTGC
	TI201-GR as NT1-R	tttgaagacaaggcagaagcTTTTTGATGAAACAGAAGC
G7_pTEI041	TI199-ER as NT1 PCR-F	tttgaagacatccATGTCTGCTGGAGACATG
	TI200-ER as NT1 PCR-R	tttgaagacaaCATtgcggcagaagcGACTGTGGCAGGGAAACC
	TI205-SDM-XVE-F	TCCTGATGATTGGcCTCGTCTGGCGCT
	TI206-SDM-XVE-R	AGCGCCAGACGAGgCCAATCATCAGGA
G8_pCK025	JS1446_ERasCT1-R	tttgaagacatAAGCCTAGACTGTGGCAGGGAAAC
	JS1447_ERasCT1-F	tttgaagacatTtcggctgcaTCTGCTGGAGACATGAGAG
G9_pAGM16841	Stonos1	tt gaagac aa CTCA ttcgtagtcaagcagatcgttcaaac
	Stonos3	tt gaagac aa CTCG agcgtcgatctagtaacatagatg
G10_pJOG173	JS967_FP_BpiI_CDS1_F	tttgaagacaaaATGGTGAGCAAGGGCGAGG
	JS968_FP_BpiI_CDS1_R	tttgaagacAAaagcTCATTTGTACAGCTCGTCCATGC
G11_pJOG172	JS967_FP_BpiI_CDS1_F	tttgaagacaaaATGGTGAGCAAGGGCGAGG
	JS968_FP_BpiI_CDS1_R	tttgaagacAAaagcTCATTTGTACAGCTCGTCCATGC
G12_pJOG171	JS967_FP_BpiI_CDS1_F	tttgaagacaaaATGGTGAGCAAGGGCGAGG
	JS968_FP_BpiI_CDS1_R	tttgaagacAAaagcTCATTTGTACAGCTCGTCCATGC
H1_pJOG021	JS860_hpt_F	TTTgaagacAAaATGAAAAAGCCTGAACTCAC
	JS861_hpt_R	TTTgaagacAAaagcCTATTCCTTTGCCCTCGGA
H2_pJOG351	JS1092_nptII_L0_F	tttgaagacaaaATGATTGAACAAGATGGATTG
	JS1093_nptII_L0_R	tttgaagacaaaagcTCAGAAGAACTCGTCAAGAAG
H3_pJOG031	JS858_AvrRps4_CDS1ns_F	tttGAAGACaaAATGACTCGAATTTCAACCAG
	JS859_AvrRps4_CDS1ns_R	TTTgaagacAAcgaagcTTGGTTGATTCTGCGaTCTTCG
H4_pJOG536	JS1126_INTACT_NTF_F	tttgaagacaaaATGGATCATTCAGCGAAAACCAC
	JS1127_INTACT_NTF_R	tttgaagacaaaagctaAGATCCACCAGTATCCTC
H5_pJOG545	Assembly from TALE Kit	n/a
H6_pCK039	Assembly from TALE Kit	n/a
H7_pCK038	Assembly from TALE Kit;	
	JS1522_TALE-Cter-F	tttggtctcaacatTCGGCCCGACCCTGCTTTAG
	JS1523_TALE-Cter-R	tttggtctcaacaacgaACCCTGTGGCAGTAACTCC
H8_pJOG641	JS1335_GVG_F	tttgaagacaaaATGGCAGATCCAATGAAGCTACTaTCTTC
	JS1336_GVG_R	tttgaagacaaaagcTCATTTTTGATGAAACAGAAG
H9_pTEI039	TI191-XVE Teil1 as CDS1-F	tttgaagacatAATGAAAGCGTTAACGGC
	TI192-XVE Teil1 as CDS1-R	tttgaagactaGgGACCAATCATCAGGATCTC
	TI193-XVE Teil2 as CDS1-F	tttgaagacatTCcCGTCTGGCGCTCCATGGAG
	TI194-XVE Teil2 as CDS1-R	tttgaagacataagCTCAGACTGTGGCAGGGAA
H10_pCK026	JS1417_Bs3F	tttgaagacataATGATGAATCAGAATTGCTT
	JS1418_Bs3DR	tttgaagacatAcAGACCATCCTCCCCCTTC
	JS1419_Bs3F2	tttgaagacatCTgTATGCAGTTGGATTTACAG
	JS1420_Bs3R2	tttgaagactaaagcCTACATTTGTTCTTTCCAAATTTTGG
H11_pJOG822	JS967_FP_BpiI_CDS1_F	tttgaagacaaaATGGTGAGCAAGGGCGAGG
	JS1184_YFP-NLS-R	tttgaagacaaaagcTTATCCTCCAACCTTTCTC
H12_pJOG587	JS1271_GUS_F	tttgaagacaaaggtTTACGTCCTGTAGAAACCC
	JS983_GUS_CDS1_R	tttgaagacaaaagcTCATTGTTTGCCTCCCTGC
pJOG947	JS1649_GW_NT1-CDS1nsF	ttgaagacatctcaCCATACAAGTTTGTACAAAAAAGC
	JS1650_GW_NT1-CDS1nsR	ttgaagactactcgCgaAACCACTTTGTACAAGAAAGCTG
pJOG956	JS1649_GW_NT1-CDS1nsF	ttgaagacatctcaCCATACAAGTTTGTACAAAAAAGC
	JS1651_GW_NT1-CDS1R	ttgaagactactcgaagcAACCACTTTGTACAAGAAAGCTG
