# Supplementary material for: Peripheral infrastructure vectors and an extended set of plant parts for the Modular Cloning system
Source: PLoS One. 2018 May 30;13(5):e0197185. doi: 10.1371/journal.pone.0197185 (PMC5976141; doi:10.1371/journal.pone.0197185)
Supplement: S2 Table — (PDF) [file pone.0197185.s007.pdf]

Table S2: Modular cloning-compatible vectors for specialized applications

GG-GW entry / MoClo->GW shuttle vectors:

name	for module type	description[1]	selection[2]	plate position
pJOG130	CDS1	attL1-[AATG-BsaI-ccdb/cat-BsaI-GCTT]-attL2	Kan, *ccdB*	A1
pJOG131	CDS1ns	attL1-[AATG-BsaI-ccdb/cat-BsaI-TTCG]-attL2	Kan, *ccdB*	A2

MoClo modules for Gateway destination vector construction:

name	for module type	description	selection	plate position
pJOG387	CDS1	BsaI_AATG[attR1-ccdB/cat-attR2]GCTT_BsaI	Spec, *ccdB*	A3
pJOG267	CDS1ns	BsaI_AATG[attR1-ccdB/cat-attR2]TTCG_BsaI	Spec, *ccdB*	A4
pJOG562	Pro->CDS1ns	BsaI_GGAG[attR1-ccdB/cat-attR2]TTCG_BsaI	Kan, *ccdB*	A5
pJOG947[3]	NT1->CDS1ns	BsaI_CCAT[attR1-ccdB/cat-attR2]TTCG_BsaI	Spec, ccdB	n/a
pJOG956[3]	NT1->CDS1	BsaI_CCAT[attR1-ccdB/cat-attR2]GCTT_BsaI	Spec, ccdB	n/a

Y2H vectors:

name	for module type	description	selection	plate position
pJOG417	CDS1	pGAD derivative; pADH1:NLS$_{SV40}$-AD$_{GAL4}$-HA-[AATG-BsaI_lacZ_BsaI-GCTT]-tADH	Amp, lacZ	A6
pJOG418	CDS1	pGBK derivative; pADH1:DBD$_{GAL4}$-myc-[AATG-BsaI_lacZ_BsaI-GCTT]-tADH	Kan, lacZ	A7
pCK011	CDS1ns	pGAD derivative; pADH1:NLS$_{SV40}$-AD$_{GAL4}$-HA-[AATG-BsaI_ccdB-cat(Cm^R)_BsaI-TTCG|TGA]-tADH	Amp, *ccdB*	A8
pCK012	CDS1ns	pGBK derivative; pADH1:DBD$_{GAL4}$-myc-[AATG-BsaI_lacZ_BsaI-TTCG|TGA]-tADH	Kan, *ccdB*	A9

Bacterial type-III secretion plasmids:

name	for module type	description	selection	plate position
pCK013	CDS1	plac:AvrRps4$_{1\text{-}134}$[AATG-BsaI_ccdB/cat_BsaI-GCTT]	Gent, *ccdB*	A10
pCK014	CDS1ns	plac:AvrRps4$_{1\text{-}134}$[AATG-BsaI_ccdB/cat_BsaI-TTCG]3xmyc	Gent, *ccdB*	A11
pCK015	CDS1	plac:AvrRpt2$_{1\text{-}100}$[AATG-BsaI_ccdB/cat_BsaI-GCTT]	Gent, *ccdB*	A12
pCK016	CDS1ns	plac:AvrRpt2$_{1\text{-}100}$[AATG-BsaI_ccdB/cat_BsaI-TTCG]3xmyc	Gent, *ccdB*	B1

Virus-induced gene silencing vector:

**name**	description	selection	plate position
pTRV2-GG	p35S:TRV-RNA2(5')[TATG-BsaI_ccdb/cat-BsaI-GGTG]RNA2(3')	Spec, *ccdB*	B2

[1] – fragment indicated as [___] is exchanged for a respective insert during Golden Gate cloning

[2] – Amp = Ampicillin or Carbenicillin; Kan = Kanamycin; Gent – Gentamicin; Spec = Spectinomycin; *ccdB* = presence of *ccdB* cassette, resistance to chloramphenicol, propagation in *ccdB* survival cells or strain DB3.1 required, lacZ = lacZ as marker gene, blue-white selection.

[3] – these modules are not contained in Addgene Kit # 1000000135.
