# Supplementary material for: Peripheral infrastructure vectors and an extended set of plant parts for the Modular Cloning system
Source: PLoS One. 2018 May 30;13(5):e0197185. doi: 10.1371/journal.pone.0197185 (PMC5976141; doi:10.1371/journal.pone.0197185)
Supplement: S3 Table — (PDF) [file pone.0197185.s008.pdf]

Table S3: Modular Cloning Level 0 modules

All plasmids carry a Spectinomycin resistance gene as selection marker. Module type refers to the 4 bp overhangs created by *Bsa*I excision as according to Engler et al. 2014 [1].

plasmid name	module type	description	plate pos.
**Promoter modules: Pro, Pro+5U and Pro+5U(f)**			
pTEI036[GB]	Pro+5U	Fusion of LexA operator and 35S minimal promoter; inducible by the synthetic TF XVE (Estradiol-inducible) [2]	B3
pTEI042[GB]<br>pJOG644[GB]	Pro+5U<br>Pro+5U(f)	Fusion of $GAL4_{UAS}$ and 35S minimal promoter; inducible by the synthetic TF GVG (Dex-inducible) [3]	B4<br>B5
pTEI038[GB]	Pro+5U	Promoter G10-90 (strong, constitutive in Arabidopsis) [4]	B6
pJOG640[GB]	Pro+5U(f)	~ 1300 bp fragment of the 35S promoter (Cauliflower mosaic virus) and 5'UTR Ω (Tobacco Mosaic Virus) [5-7]	B7
pJOG684*[GB]	Pro+5U	~ 800 bp fragment upstream of Arabidopsis *UBQ10* (At4g05320) (strong, constitutive promoter) [8]	B8
pJOG603<br>pTEI044	Pro+5U<br>Pro+5U(f)	~ 1700 bp upstream of Arabidopsis *RPS5a* (At3g11940) (strong, constitutive promoter in Arabidopsis) [9]	B9<br>B10
pJOG301	Pro+5U	~ 1000 bp upstream of Arabidopsis *DD45/EC1.2* (At2g21740) (egg cell-specific promoter) [10, 11]	B11
pAGM28521[GB]	Pro+5U	Egg cell-specific promoter: Fusion of the *EC1.2* enhancer with *EC1.1* promoter (*EC1.2en-EC1.1p*) [11]	B12
pJOG299	Pro+5U	~ 1400 bp upstream of Arabidopsis *AP1* (At1g69120) (flower mersitem-specific promoter) [12]	C1
pJOG298[GB]	Pro+5U	~ 600 bp fragment upstream of Arabidopsis *ICU2* (At5g67100) (dividing cell-specific promoter) [13]	C2
pJOG295*[GB]	Pro+5U	~ 2100 bp fragment upstream of Arabidopsis *GILT* (At4g12960)	C3
pJOG300*[GB]	Pro+5U	~ 350 bp fragment upstream of Arabidopsis At1g66850	C4
pJOG025<br>pJOG123	Pro+5U<br>Pro+5U(f)	~ 1000 bp fragment upstream of Arabidopsis *EDS1* (At3g48090) [14]	C5<br>C6
pJOG026<br>pJOG124	Pro+5U<br>Pro+5U(f)	~1200 bp fragment upstream of Arabidopsis *PAD4* (At3g52430) [15, 16]	C7<br>C8
pJOG466[GB]<br>pJOG467[GB]	Pro+5U<br>Pro+5U(f)	~ 1000 bp fragment upstream of Arabidopsis *SAG101* (At5g14920) [17]	C9<br>C10
pJOG010[GB]	Pro+5U	~ 1200 bp fragment upstream of Arabidopsis *PR1* (At2g14610) (pathogen-responsive promoter) [18]	C11
pJOG648	Pro+5U	~ 550 bp fragment upstream of Arabidopsis *SESA3/AtS2S3* (At4g27160) (seed-specific promoter) [19]	C12
pTEI067*[GB]	Pro+5U	~ 1500 bp fragment upstream of Arabidopsis *CER6* (At1g68530) (epidermis-specific promoter) [20]	D1
pTEI068*[GB]	Pro+5U	~ 2000 bp fragment upstream of Arabidopsis *CUE1* (At5g33320) (mesophyll-specific promoter) [21]	D2
pTEI069[GB]	Pro+5U	~ 1000 bp fragment upstream of Arabidopsis *BODYGUARD1* (At1g64670) (epidermis-specific promoter) [22]	D3
pTEI070[GB]	Pro+5U	~ 1000 bp fragment upstream of Arabidopsis At1g22690 ("*pGC1*") (guard cell-specific promoter) [23]	D4
pTEI071[GB]	Pro+5U	~ 1000 bp fragment upstream of Arabidopsis *CAB3* (At1g29910) (mesophyll-specific promoter) [24]	D5
pTEI073*[GB]	Pro+5U	~ 2000 bp fragment upstream of Arabidopsis *ALMT12* (At4tg17585) (guard cell-specific promoter) [25]	D6
pCK019*[GB]	Pro+5U	~ 750 bp fragment upstream of Arabidopsis *EXP7* (At1g12560) (root hair-specific promoter) [26, 27]	D7
pCK020*	Pro+5U	~ 2300 bp fragment upstream of Arabidopsis *WOX5* (At3g11260) (root stem cell niche / quiescent center-specific promoter) [28]	D8

plasmid name	module type	description	plate pos.
**Promoter modules: Pro, Pro+5U and Pro+5U(f) [continued]**			
pCK021*[GB]	Pro+5U	~ 3300 bp fragment upstream of Arabidopsis *SOMBRERO* (At1g79580) (root cap-specific promoter) [29, 30]	D9
pAGM1761[GB]	Pro	~ 3000 bp fragment upstream of Arabidopsis *AtDMC1* (At3G22880) (meiosis-specific promoter) [31]	D10
pJOG020[GB]<br>pJOG575[GB]	Pro+5U<br>Pro+5U(f)	~ 1000 bp fragment upstream of parsley *Ubi4-2* [32]	D11<br>D12
pJOG022[GB]	Pro+5U	~ 1500 bp fragment of the *Zea mays* Ubiquitin promoter + UTR [33]	E1
pJOG213[GB]<br>pJOG004[GB]	Pro+5U<br>Pro+5U(f)	~ 1200 bp fragment upstream of *SlEDS1* (Solyc060g071280)	E2<br>E3
pJOG535[GB]<br>pJOG577[GB]	Pro+5U<br>Pro+5U(f)	~ 1000 bp fragment of the *CaBs3* promoter (TALE = AvrBs3-inducible promoter) [34]	E4<br>E5
pJOG638[GB]<br>pJOG639[GB]	Pro+5U<br>Pro+5U(f)	~ 1000 bp fragment of the *CaBs3* promoter (TALE = AvrBs3-inducible promoter); weaker variant; mutation in TALE-binding site	E6<br>E7

plasmid name	module type	description	plate pos.
**Tag modules: NT1 and CT**			
pJOG170[GB]<br>pJOG176[GB]	NT1<br>CT	CDS of mEGFP variant of GFP (*Aequorea victoria*) [35, 36]	E8<br>E9
pJOG169[GB]<br>pJOG175[GB]	NT1<br>CT	CDS of mTRQ variant of GFP (*Aequorea victoria*) [35, 37]	E10<br>E11
pJOG168[GB]<br>pJOG174[GB]	NT1<br>CT	CDS of mVenus variant of GFP (*Aequorea victoria*) [35, 38]	E12<br>F1
pJOG142[GB]	CT	mYFP-NES; CDS of mYFP fused to the PKI nuclear export signal [39, 40]	F2
pJOG143[GB]	CT	mYFP-NLS; CDS of mYFP fused to SV40 nuclear localization signal [40, 41]	F3
pJOG140[GB]	NT1	NES; PKI nuclear export signal [39]	F4
pJOG141[GB]<br>pJOG824[GB]	NT1<br>CT	NLS; SV40 nuclear localization signal [41]	F5<br>F6
pAGM16456[GB]	NT1	6xHis-NES(Rev); fusion of 6xHis tag with nuclear export signal (NES from Rev, HIV-1) [39]	F7
pAGM16467[GB]	NT1	6xHis-NES(PKI); fusion of 6xHis tag with nuclear export signal (NES from PKI) [39]	F8
pAGM16418*[GB]	NT1	6xHis-NES(Rev)-GFP; fusion of 6xHis tag with NES (Rev) and GFP	F9
pAGM16429*[GB]	NT1	6xHis-NES(PKI)-GFP ; fusion of 6xHis tag with NES (PKI) and GFP	F10
pJOG642[GB]	NT1	3 x HA tag fused to the tagRFP-T CDS [42]	F11
pJOG317[GB]	CT	4 x myc tag fused to Twin-Strep tag [43]	F12
pJOG331[GB]	CT	6 x HA tag fused to Twin-Strep tag [43]	G1
pJOG658[GB]	NT1	3 x HA tag fused to firefly (*Photinus pyralis*) Luciferase CDS [44]	G2
pJOG659[GB]	CT	Renilla (*Renilla reniformis*) luciferase CDS fused to a 3 x HA tag [45]	G3
pJOG643[GB]<br>pCK032[GB]	NT1<br>CT	Hormone-binding domain of the rat glucocorticoid receptor [3, 46]	G4<br>G5
pTEI037*[GB]	NT1	Hormone-binding domain of the rat glucocorticoid receptor fused to mEGFP [3, 36]	G6
pTEI041<br>pCK025	NT1<br>CT	Hormone-binding domain and regulatory sequences of the human estrogen receptor [2, 47]	G7<br>G8

plasmid name	module type	description	plate pos.
**STOP module: CT -> 3U+ter**			
pAGM16841[GB]	CT-3Uter	STOP codon and *nos* terminator; to fuse a STOP codon to CDS1ns modules while simultaneously adding a *nos* terminator	G9

plasmid name	module type	description	plate pos.
**CDS modules: CDS1, CDS1ns and CDS2**			
pJOG173[GB]	CDS1	CDS of mEGFP variant of GFP (*Aequorea victoria*) [35, 36]	G10
pJOG172[GB]	CDS1	CDS of mTRQ variant of GFP (*Aequorea victoria*) [35, 37]	G11
pJOG171[GB]	CDS1	CDS of mVenus variant of GFP (*Aequorea victoria*) [35, 38]	G12
pJOG021	CDS1	Hygromycin phosphotransferase (hpt) CDS (*Escherichia coli*) [48]	H1
pJOG351[GB]	CDS1	Neomycin phosphotransferase (nptII) CDS (*Escherichia coli*) [48]	H2
pJOG031	CDS1	AvrRps4 (*Pseudomonas syringae* pv. *pisi*) [49]	H3
pJOG536*	CDS1	"Nuclear targeting factor" [RanGAP1(WPP)-GFP-BLRP] of the INTACT system [50]	H4
pJOG545	CDS1	AvrBs3 (*Xanthomonas campestris* pv. *vesicatoria*) [51]	H5
pCK039*[GB]<br>pCK038*[GB]	CDS1<br>CDS1ns	dTALE binding to Bs3 promoter (*Capsicum annuum*) as transactivator	H6<br>H7
pJOG641[GB]	CDS1	GVG synthetic TF: GAL4-VP16-GR (inducible expression, dexamethasone) [3]	H8
pTEI039	CDS1	XVE synthetic TF: LexA-VP16-ER (inducible expression, estradiol) [2]	H9
pCK026[GB]	CDS1	*Bs3* gene (encodes an FMO enzyme) from *Capsicum annuum* [34]	H10
pJOG822[GB]	CDS1	Yellow Fluorescent Protein *(YFP)* fused with SV40 NLS	H11
pJOG587[GB]	CDS2	β-glucuronidase (*Escherichia coli*) CDS with two introns, no ATG-START codon [1]	H12

* module not experimentally validated

[GB] comaptible with GoldenBraid system (no *BsmB*I sites)

	1	2	3	4	5	6	7	8	9	10	11	12
A	pJOG130<br>CDS1<br>GG-GW<br>shuttle	pJOG131<br>CDS1ns<br>GG-GW<br>shuttle	pJOG387<br>CDS1<br>GW<br>destination	pJOG267<br>CDS1ns<br>GW<br>destination	pJOG562<br>Pro+CDS1ns<br>GW<br>destination	pJOG417<br>CDS1<br>Y2H-AD	pJOG418<br>CDS1<br>Y2H-BD	pCK011<br>CDS1ns<br>Y2H-AD	pCK012<br>CDS1ns<br>Y2H-BD	pCK013<br>CDS1<br>Bacterial<br>secretion<br>AvrRps4	pCK014<br>CDS1ns<br>Bacterial<br>secretion<br>AvrRps4	pCK015<br>CDS1<br>Bacterial<br>secretion<br>AvrRpt2
B	pCK016<br>CDS1ns<br>Bacterial<br>secretion<br>AvrRpt2	pTRV2-GG<br>VIGS<br>GG-TRV2	pTEI036<br>Pro+5U<br>min35S:LexA<br>Estradiol<br>inducible	pTEI042<br>Pro+5U<br>min35S:<br>GAL4$_{UAS}$<br>DEX-<br>inducible	pJOG644<br>Pro+5U(f)<br>min35S:<br>GAL4$_{UAS}$<br>DEX-<br>inducible	pTEI038<br>Pro+5U<br>pG10-90	pJOG640<br>Pro+5U(f)<br>35S:+5'UTR<br>of TMV	pJOG684<br>Pro+5U<br>p(At)*UBQ10*	pJOG603<br>Pro+5U<br>p(*At*)RPS5a	pTEI044<br>Pro+5U(f)<br>p(*At*)RPS5a	pJOG301<br>Pro+5U<br>p(*At*)*DD45/*<br>*EC1.2*	pAGM28521<br>Pro+5U<br>fusion<br>p(*At*)*EC1.2en*<br>*+EC1.1*
C	pJOG299<br>Pro+5U<br>p(*At*)*AP1*	pJOG298<br>Pro+5U<br>p(*At*)*ICU2*	pJOG295<br>Pro+5U<br>p(*At*)*GILT*	pJOG300<br>Pro+5U<br>pAt1G66850	pJOG025<br>Pro+5U<br>p(*At*)*EDS1*	pJOG123<br>Pro+5U(f)<br>p(*At*)*EDS1*	pJOG026<br>Pro+5U<br>p(*At*)*PAD4*	pJOG124<br>Pro+5U(f)<br>p(*At*)*PAD4*	pJOG466<br>Pro+5U<br>p(*At*)*SAG101*	pJOG467<br>Pro+5U(f)<br>p(*At*)*SAG101*	pJOG010<br>Pro+5U<br>p(*At*)*PR1*	pJOG648<br>Pro+5U<br>p(*At*)*SESA3*
D	pTEI067<br>Pro+5U<br>p(*At*)*CER6*	pTEI068<br>Pro+5U<br>p(*At*)*CUE1*	pTEI069<br>Pro+5U<br>p(*At*)*BODY-*<br>*GUARD1*	pTEI70<br>Pro+5U<br>p(*At*)*GC1*	pTEI071<br>Pro+5U<br>p(*At*)*CAB3*	pTEI073<br>Pro+5U<br>p(*At*)*ALMT12*	pCK019<br>Pro+5U<br>p(*At*)*EXP7*	pCK020<br>Pro+5U<br>p(*At*)*WOX5*	pCK021<br>Pro+5U<br>p(*At*)*SOM-*<br>*BRERO*	pAGM1761<br>Pro<br>p(*At*)DMC1	pJOG020<br>Pro+5U<br>p(*Pc*)*Ubi4-2*	pJOG575<br>Pro+5U(f)<br>p(*Pc*)*Ubi4-2*
E	pJOG022<br>Pro+5U<br>p(Zm)Ubi	pJOG213<br>Pro+5U<br>p(*Sl*)*EDS1*	pJOG004<br>Pro+5U(f)<br>p(*Sl*)*EDS1*	pJOG535<br>Pro+5U<br>p(*Ca*)*Bs3*	pJOG577<br>Pro+5U(f)<br>p(*Ca*)*Bs3*	pJOG638<br>Pro+5U<br>p(*Ca*)*Bs3*<br>weaker	pJOG639<br>Pro+5U(f)<br>p(*Ca*)*Bs3*<br>weaker	pJOG170<br>NT1<br>CDS of<br>mEGFP	pJOG176<br>CT<br>CDS of<br>mEGFP	pJOG169<br>NT1<br>CDS of<br>mTRQ	pJOG175<br>CT<br>CDS of<br>mTRQ	pJOG168<br>NT1<br>CDS of<br>mVenus
F	pJOG174<br>CT<br>CDS of<br>mVenus	pJOG142<br>CT<br>mYFP-NES	pJOG143<br>CT<br>mYFP-NLS	pJOG140<br>NT1<br>NES	pJOG141<br>NT1<br>NLS	pJOG824<br>CT<br>NLS	pAGM16456<br>NT1<br>6xHis-NES<br>(Rev)	pAGM16467<br>NT1<br>6xHis-NES<br>(PKI)	pAGM16418<br>NT1<br>6xHis-<br>NES(Rev)-<br>GFP	pAGM16429<br>NT1<br>6xHis-<br>NES(PKI)-<br>GFP	pJOG642<br>NT1<br>3xHA-<br>tagRFP-T	pJOG317<br>CT<br>4 x Myc-<br>2Strep
G	pJOG331<br>CT<br>6xHA-<br>2Strep	pJOG658<br>NT1<br>3xHA-LUC	pJOG659<br>CT<br>REN-3xHA	pJOG643<br>NT1<br>GR (rat)	pCK032<br>CT<br>GR (rat)	pTEI037<br>NT1<br>GR-mEGFP	pTEI041<br>NT1<br>ER (human)	pCK025<br>CT<br>ER (human)	pAGM16841<br>CT-3U+ter<br>STOP+*nos*<br>terminator	pJOG173<br>CDS1<br>mEGFP	pJOG172<br>CDS1<br>mTRQ	pJOG171<br>CDS1<br>mVenus
H	pJOG021<br>CDS1<br>hpt	pJOG351<br>CDS1<br>nptII	pJOG031<br>CDS1<br>AvrRps4	pJOG536<br>CDS1<br>NTF from<br>INTACT	pJOG545<br>CDS1<br>AvrBs3	pCK039<br>CDS1<br>dTALE	pCK038<br>CDS1ns<br>dTALE	pJOG641<br>CDS1<br>GVG<br>synthetic TF	pTEI039<br>CDS1<br>XVE<br>synthetic TF	pCK026<br>CDS1<br>*Bs3*	pJOG822<br>CDS1<br>*YFP*-NLS	pJOG587<br>CDS2<br>GUS-ATG

contains *ccdB /cat* cassette. Propagation of empty plasmid requires ccdB survival/DB3.1 cells, and additional Chloramphenicol selection is advised.

contains *lacZ* fragment for blue/white selection

bacterial selection: Kanamycin

bacterial selection: Spectinomycin

bacterial selection: Ampicillin / Carbenicillin

bacterial selection: Gentamicin

References

1. Engler, C., M. Youles, R. Gruetzner, T.M. Ehnert, S. Werner, J.D. Jones, N.J. Patron, and S. Marillonnet, *A Golden Gate Modular Cloning Toolbox for Plants.* ACS Synth Biol, 2014.
2. Zuo, J., Q.W. Niu, and N.H. Chua, *An estrogen receptor-based transactivator XVE mediates highly inducible gene expression in transgenic plants.* Plant J, 2000. **24**(2): p. 265-73.
3. Aoyama, T. and N.H. Chua, *A glucocorticoid-mediated transcriptional induction system in transgenic plants.* Plant J, 1997. **11**(3): p. 605-12.
4. Ishige, F., M. Takaichi, R. Foster, N.H. Chua, and K. Oeda, *A G-box motif (GCCACGTGCC) tetramer confers high-level constitutive expression in dicot and monocot plants.* Plant J, 1999. **18**(4): p. 443–448.
5. Gallie, D.R., D.E. Sleat, J.W. Watts, P.C. Turner, and T.M. Wilson, *The 5'-leader sequence of tobacco mosaic virus RNA enhances the expression of foreign gene transcripts in vitro and in vivo.* Nucleic Acids Res, 1987. **15**(8): p. 3257-73.
6. Ow, D.W., J.D. Jacobs, and S.H. Howell, *Functional regions of the cauliflower mosaic virus 35S RNA promoter determined by use of the firefly luciferase gene as a reporter of promoter activity.* PNAS, 1987. **84**(14): p. 4870-4.
7. Guilley, H., R.K. Dudley, G. Jonard, E. Balazs, and K.E. Richards, *Transcription of Cauliflower mosaic virus DNA: detection of promoter sequences, and characterization of transcripts.* Cell, 1982. **30**(3): p. 763-73.
8. Grefen, C., N. Donald, K. Hashimoto, J. Kudla, K. Schumacher, and M.R. Blatt, *A ubiquitin-10 promoter-based vector set for fluorescent protein tagging facilitates temporal stability and native protein distribution in transient and stable expression studies.* Plant J, 2010. **64**(2): p. 355-65.
9. Tsutsui, H. and T. Higashiyama, *pKAMA-ITACHI Vectors for Highly Efficient CRISPR/Cas9-Mediated Gene Knockout in Arabidopsis thaliana.* Plant Cell Physiol, 2017. **58**(1): p. 46-56.
10. Steffen, J.G., I.H. Kang, J. Macfarlane, and G.N. Drews, *Identification of genes expressed in the Arabidopsis female gametophyte.* Plant J, 2007. **51**(2): p. 281-92.
11. Wang, Z.P., H.L. Xing, L. Dong, H.Y. Zhang, C.Y. Han, X.C. Wang, and Q.J. Chen, *Egg cell-specific promoter-controlled CRISPR/Cas9 efficiently generates homozygous mutants for multiple target genes in Arabidopsis in a single generation.* Genome Biol, 2015. **16**: p. 144.
12. Gao, Y., Y. Zhang, D. Zhang, X. Dai, M. Estelle, and Y. Zhao, *Auxin binding protein 1 (ABP1) is not required for either auxin signaling or Arabidopsis development.* PNAS, 2015. **112**(7): p. 2275-80.
13. Hyun, Y., J. Kim, S.W. Cho, Y. Choi, J.S. Kim, and G. Coupland, *Site-directed mutagenesis in Arabidopsis thaliana using dividing tissue-targeted RGEN of the CRISPR/Cas system to generate heritable null alleles.* Planta, 2015. **241**(1): p. 271-84.
14. Wagner, S., J. Stuttmann, S. Rietz, R. Guerois, E. Brunstein, J. Bautor, K. Niefind, and J.E. Parker, *Structural basis for signaling by exclusive EDS1 heteromeric complexes with SAG101 or PAD4 in plant innate immunity.* Cell Host Microbe, 2013. **14**(6): p. 619-30.
15. Feys, B.J., L.J. Moisan, M.A. Newman, and J.E. Parker, *Direct interaction between the Arabidopsis disease resistance signaling proteins, EDS1 and PAD4.* EMBO Journal, 2001. **20**(19): p. 5400-5411.
16. Jirage, D., T.L. Tootle, T.L. Reuber, L.N. Frost, B.J. Feys, J.E. Parker, F.M. Ausubel, and J. Glazebrook, *Arabidopsis thaliana PAD4 encodes a lipase-like gene that is important for salicylic acid signaling.* PNAS, 1999. **96**(23): p. 13583-8.
17. Feys, B.J., M. Wiermer, R.A. Bhat, L.J. Moisan, N. Medina-Escobar, C. Neu, A. Cabral, and J.E. Parker, *Arabidopsis SENESCENCE-ASSOCIATED GENE101 stabilizes and signals within an ENHANCED DISEASE SUSCEPTIBILITY1 complex in plant innate immunity.* Plant Cell, 2005. **17**(9): p. 2601-13.
18. van Loon, L.C., M. Rep, and C.M. Pieterse, *Significance of inducible defense-related proteins in infected plants.* Annu Rev Phytopathol, 2006. **44**: p. 135-62.
19. Kroj, T., G. Savino, C. Valon, J. Giraudat, and F. Parcy, *Regulation of storage protein gene expression in Arabidopsis.* Development, 2003. **130**(24): p. 6065-73.

20. Hooker, T.S., A.A. Millar, and L. Kunst, *Significance of the expression of the CER6 condensing enzyme for cuticular wax production in Arabidopsis.* Plant Phys, 2002. **129**(4): p. 1568-80.
21. Li, H., K. Culligan, R.A. Dixon, and J. Chory, *CUE1: A Mesophyll Cell-Specific Positive Regulator of Light-Controlled Gene Expression in Arabidopsis.* Plant Cell, 1995. **7**(10): p. 1599-1610.
22. Kurdyukov, S., A. Faust, C. Nawrath, S. Bar, D. Voisin, N. Efremova, R. Franke, L. Schreiber, H. Saedler, J.P. Metraux, and A. Yephremov, *The epidermis-specific extracellular BODYGUARD controls cuticle development and morphogenesis in Arabidopsis.* Plant Cell, 2006. **18**(2): p. 321-39.
23. Yang, Y., A. Costa, N. Leonhardt, R.S. Siegel, and J.I. Schroeder, *Isolation of a strong Arabidopsis guard cell promoter and its potential as a research tool.* Plant Methods, 2008. **4**: p. 6.
24. Susek, R.E., F.M. Ausubel, and J. Chory, *Signal transduction mutants of Arabidopsis uncouple nuclear CAB and RBCS gene expression from chloroplast development.* Cell, 1993. **74**(5): p. 787-99.
25. Meyer, S., P. Mumm, D. Imes, A. Endler, B. Weder, K.A. Al-Rasheid, D. Geiger, I. Marten, E. Martinoia, and R. Hedrich, *AtALMT12 represents an R-type anion channel required for stomatal movement in Arabidopsis guard cells.* Plant J, 2010. **63**(6): p. 1054-62.
26. Stenzel, I., T. Ischebeck, S. Konig, A. Holubowska, M. Sporysz, B. Hause, and I. Heilmann, *The type B phosphatidylinositol-4-phosphate 5-kinase 3 is essential for root hair formation in Arabidopsis thaliana.* Plant Cell, 2008. **20**(1): p. 124-41.
27. Cho, H.T. and D.J. Cosgrove, *Regulation of root hair initiation and expansin gene expression in Arabidopsis.* Plant Cell, 2002. **14**(12): p. 3237-53.
28. Sarkar, A.K., M. Luijten, S. Miyashima, M. Lenhard, T. Hashimoto, K. Nakajima, B. Scheres, R. Heidstra, and T. Laux, *Conserved factors regulate signalling in Arabidopsis thaliana shoot and root stem cell organizers.* Nature, 2007. **446**(7137): p. 811-4.
29. Bennett, T., A. van den Toorn, G.F. Sanchez-Perez, A. Campilho, V. Willemsen, B. Snel, and B. Scheres, *SOMBRERO, BEARSKIN1, and BEARSKIN2 regulate root cap maturation in Arabidopsis.* Plant Cell, 2010. **22**(3): p. 640-54.
30. Willemsen, V., M. Bauch, T. Bennett, A. Campilho, H. Wolkenfelt, J. Xu, J. Haseloff, and B. Scheres, *The NAC domain transcription factors FEZ and SOMBRERO control the orientation of cell division plane in Arabidopsis root stem cells.* Dev Cell, 2008. **15**(6): p. 913-22.
31. Klimyuk, V.I. and J.D. Jones, *AtDMC1, the Arabidopsis homologue of the yeast DMC1 gene: characterization, transposon-induced allelic variation and meiosis-associated expression.* Plant J, 1997. **11**(1): p. 1-14.
32. Fauser, F., S. Schiml, and H. Puchta, *Both CRISPR/Cas-based nucleases and nickases can be used efficiently for genome engineering in Arabidopsis thaliana.* Plant J, 2014. **79**(2): p. 348-59.
33. Christensen, A.H., R.A. Sharrock, and P.H. Quail, *Maize polyubiquitin genes: structure, thermal perturbation of expression and transcript splicing, and promoter activity following transfer to protoplasts by electroporation.* Plant Mol Biol, 1992. **18**(4): p. 675-89.
34. Romer, P., S. Hahn, T. Jordan, T. Strauss, U. Bonas, and T. Lahaye, *Plant pathogen recognition mediated by promoter activation of the pepper Bs3 resistance gene.* Science, 2007. **318**(5850): p. 645-8.
35. Hecker, A., N. Wallmeroth, S. Peter, M.R. Blatt, K. Harter, and C. Grefen, *Binary 2in1 Vectors Improve in Planta (Co)localization and Dynamic Protein Interaction Studies.* Plant Phys, 2015. **168**(3): p. 776-87.
36. Yang, T.T., L. Cheng, and S.R. Kain, *Optimized codon usage and chromophore mutations provide enhanced sensitivity with the green fluorescent protein.* Nucleic Acids Res, 1996. **24**(22): p. 4592-3.
37. Goedhart, J., D. von Stetten, M. Noirclerc-Savoye, M. Lelimousin, L. Joosen, M.A. Hink, L. van Weeren, T.W. Gadella, Jr., and A. Royant, *Structure-guided evolution of cyan fluorescent proteins towards a quantum yield of 93%.* Nat Commun, 2012. **3**: p. 751.

38. Nagai, T., K. Ibata, E.S. Park, M. Kubota, K. Mikoshiba, and A. Miyawaki, *A variant of yellow fluorescent protein with fast and efficient maturation for cell-biological applications.* Nature Biotechnology, 2002. **20**(1): p. 87-90.
39. Wen, W., J.L. Meinkoth, R.Y. Tsien, and S.S. Taylor, *Identification of a signal for rapid export of proteins from the nucleus.* Cell, 1995. **82**(3): p. 463-73.
40. Garcia, A.V., S. Blanvillain-Baufume, R.P. Huibers, M. Wiermer, G. Li, E. Gobbato, S. Rietz, and J.E. Parker, *Balanced nuclear and cytoplasmic activities of EDS1 are required for a complete plant innate immune response.* PLoS Pathog, 2010. **6**: p. e1000970.
41. Lange, A., R.E. Mills, C.J. Lange, M. Stewart, S.E. Devine, and A.H. Corbett, *Classical nuclear localization signals: definition, function, and interaction with importin alpha.* J Biol Chem, 2007. **282**(8): p. 5101-5.
42. Shaner, N.C., M.Z. Lin, M.R. McKeown, P.A. Steinbach, K.L. Hazelwood, M.W. Davidson, and R.Y. Tsien, *Improving the photostability of bright monomeric orange and red fluorescent proteins.* Nat Methods, 2008. **5**(6): p. 545-51.
43. Schmidt, T.G., L. Batz, L. Bonet, U. Carl, G. Holzapfel, K. Kiem, K. Matulewicz, D. Niermeier, I. Schuchardt, and K. Stanar, *Development of the Twin-Strep-tag(R) and its application for purification of recombinant proteins from cell culture supernatants.* Protein Expr Purif, 2013. **92**(1): p. 54-61.
44. Gould, S.J. and S. Subramani, *Firefly luciferase as a tool in molecular and cell biology.* Anal Biochem, 1988. **175**(1): p. 5-13.
45. Lorenz, W.W., M.J. Cormier, D.J. O'Kane, D. Hua, A.A. Escher, and A.A. Szalay, *Expression of the Renilla reniformis luciferase gene in mammalian cells.* J Biolumin Chemilumin, 1996. **11**(1): p. 31-7.
46. Picard, D., *Steroid-binding domains for regulating the functions of heterologous proteins in cis.* Trends Cell Biol, 1993. **3**(8): p. 278-80.
47. Greene, G.L., P. Gilna, M. Waterfield, A. Baker, Y. Hort, and J. Shine, *Sequence and expression of human estrogen receptor complementary DNA.* Science, 1986. **231**(4742): p. 1150-4.
48. Sundar, I.K. and N. Sakthivel, *Advances in selectable marker genes for plant transformation.* J Plant Phys, 2008. **165**(16): p. 1698-716.
49. Hinsch, M. and B. Staskawicz, *Identification of a new Arabidopsis disease resistance locus, RPS4, and cloning of the corresponding avirulence gene, avrRps4, from Pseudomonas syringae pv. pisi.* Mol Plant Microbe Interact, 1996. **9**(1): p. 55-61.
50. Deal, R.B. and S. Henikoff, *A simple method for gene expression and chromatin profiling of individual cell types within a tissue.* Dev Cell, 2010. **18**(6): p. 1030-40.
51. Boch, J. and U. Bonas, *Xanthomonas AvrBs3 family-type III effectors: discovery and function.* Annu Rev Phytopathol, 2010. **48**: p. 419-36.
